# SUMOylation of RALY promotes vasculogenic mimicry in glioma cells via the FOXD1/DKK1 pathway

**DOI:** 10.1007/s10565-023-09836-3

**Published:** 2023-10-31

**Authors:** Shuo Cao, Di Wang, Ping Wang, Yunhui Liu, Weiwei Dong, Xuelei Ruan, Libo Liu, Yixue Xue, Tiange E, Hongda Lin, Xiaobai Liu

**Affiliations:** 1 Key Laboratory of Neuro-Oncology in Liaoning Province, Shenyang, 110004 China; 2 Department of Oncology, Shengjing Hospital of China Medical University, Shenyang, 110004 Liaoning Province China; 3 Department of Neurosurgery, Shengjing Hospital of China Medical University, Shenyang, 110004 China; 4 Liaoning Medical Surgery and Rehabilitation Robot Technology Engineering Research Center, Shenyang, 110004 China; 5 Department of Neurobiology, School of Life Sciences, China Medical University, Shenyang, 110122 China

**Keywords:** UBA2, RALY, SUMOylation, FOXD1, Vasculogenic mimicry, Glioma

## Abstract

Human malignant gliomas are the most common and aggressive primary malignant tumors of the human central nervous system. Vasculogenic mimicry (VM), which refers to the formation of a tumor blood supply system independently of endothelial cells, contributes to the malignant progression of glioma. Therefore, VM is considered a potential target for glioma therapy. Accumulated evidence indicates that alterations in SUMOylation, a reversible post-translational modification, are involved in tumorigenesis and progression. In the present study, we found that UBA2 and RALY were upregulated in glioma tissues and cell lines. Downregulation of UBA2 and RALY inhibited the migration, invasion, and VM of glioma cells. RALY can be SUMOylated by conjugation with SUMO1, which is facilitated by the overexpression of UBA2. The SUMOylation of RALY increases its stability, which in turn increases its expression as well as its promoting effect on FOXD1 mRNA. The overexpression of FOXD1 promotes DKK1 transcription by activating its promoter, thereby promoting glioma cell migration, invasion, and VM. Remarkably, the combined knockdown of UBA2, RALY, and FOXD1 resulted in the smallest tumor volumes and the longest survivals of nude mice in vivo. UBA2/RALY/FOXD1/DKK1 axis may play crucial roles in regulating VM in glioma, which may contribute to the development of potential strategies for the treatment of gliomas.

**Supplementary information:**

The online version contains supplementary material available at 10.1007/s10565-023-09836-3.

## Introduction

Gliomas are the most common and lethal nervous system tumors (Lapointe et al. 2018; Gravina et al. 2017). Conventional glioma treatment comprises surgical resection, chemotherapy, radiation therapy, and combination therapy. However, despite advances in combination therapies, the treatment of high-grade gliomas is still challenging, with low therapeutic success and overall survival rates (Wick et al. 2018; Malta et al. 2018). Anti-angiogenesis therapy has become one of the current research hotspots in the treatment of glioma, but the clinical efficacy of anti-tumor angiogenesis drugs represented by bevacizumab is still not satisfactory (Szklener et al. 2022). Glioma is characterized by hypervascularization, and neovascularization promotes its malignant progression by providing essential oxygen and nutrients (Domènech et al. 2021). However, neovascularization in tumors is a highly complicated process. Besides sprouting angiogenesis, many other vascularization mechanisms, such as endothelial progenitor cell (EPC) recruitment, vascular co-option, intussusceptive angiogenesis, and VM, have been discovered in tumors (Li et al. 2023; Ayala-Dominguez et al. 2019). VM represents a tumor microcirculation model that refers to the formation of non-endothelial blood vessels by tumor cells through extracellular matrix remodeling and deformation (Fernandez-Cortes et al. 2019). The phenomenon of VM has been confirmed in a variety of tumors, including hepatocellular carcinoma (HCC), non-small-cell lung cancer (NSCLC), and glioma (Chu et al. 2021; Huang et al. 2023; Zhou et al. 2021). It is associated with aggressive biological behavior, reduced anti-angiogenesis treatment efficacy, and enhanced tumor-related mortality (Dudley et al. 2023). Targeted treatment of glioma VM has shown positive therapeutic effects in animal models (Ju et al. 2016). Therefore, further clarifying the molecular mechanism of VM in glioma might open a new perspective for glioma treatment. SUMOylation is a reversible post-translational modification, in which a small ubiquitin-like modifier (SUMO) is covalently attached to the lysine residues of the substrates. This enzymatic reaction is carried out by SUMO-activating enzyme (E1), SUMO-conjugating enzyme (E2, UBE2I), and SUMO ligase (E3) (Mo et al. 2005; Rytinki et al. 2009; Mukhopadhyay et al. 2007). There are four subtypes of SUMOs, SUMO1–4. SUMO-specific proteases, such as the most extensively studied SENP3, can remove the conjugated SUMO from the substrate protein (Yeh et al. 2009). Ubiquitin-like modifier-activating enzyme 2 (UBA2) is a subunit of E1, which is up-regulated in many tumors and functions as an oncogene by promoting the SUMOylation of proteins (Wang et al. 2020). SUMOylation is involved in many cellular activities, including nucleocytoplasmic transport, protein–protein interaction, transcriptional regulation, and genomic stability maintenance, and its alterations are associated with tumorigenesis and progression (Han et al. 2018; Lin et al. 2023; Zhao et al. 2018).

Heteronuclear ribonucleoprotein (hnRNP) is a large class of RNA-binding proteins, which plays crucial roles in the process of RNA metabolism. RALY, an hnRNP family protein, contains both a leucine-zipper coiled domain and an RNA-binding domain, suggesting that it likely functions as a regulatory factor. By binding to poly-U elements within multiple RNAs, RALY can regulate the expression of specific transcripts. As previously reported, RALY can stabilize *E2F1* mRNA via binding with its 3′-UTR (Rossi et al. 2017; Cornella et al. 2017). In HCC, RALY expression predicts poor prognosis and metastasis in HCC patients. Overexpression of RALY promotes HCC tumorigenicity, migration, and invasion. Moreover, RALY could upregulate TGF-β2 by binding to its mRNA and improving its stability (Wang et al. 2021). According to the GPS-SUMO (http://hemi.biocuckoo.org) software, RALY can be SUMOylated in cells. However, the profiling of RALY in gliomas remains unclear.

FOXD1 belongs to the forkhead family of transcription factors and regulates numerous cellular functions implicated in cancer development (Quintero-Ronderos et al. 2018; Kun et al. 2022). Recently, it has been proven that FOXD1 is significantly elevated in breast cancer cells and tissues, and it promotes migration and metastasis of BC cells by upregulating RalA via directly bound to RalA promotor (Long et al. 2022). Moreover, it shows increased expression of FOXD1 in mesenchymal glioma stem cells and contributes to glial neoplasms by upregulating the expression of the aldehyde dehydrogenase isoform ALDH1A3 via directly activating its promoter (Cheng et al. 2016). In addition, recent research has indicated that the expression of FOXD1 is increased in glioma, while its inhibition decreases cell viability and migration and promotes cell senescence in the human glioblastoma cell line (Gao et al. 2017). However, further investigation is required to explore the role of FOXD1 in the regulation of the vasculogenic mimicry in glioma cells.

DKK1 is a member of the Dickkopf family, which can hinder the β-catenin-dependent Wnt signaling pathway through its interaction with LRP5/6. In addition, DKK1 stimulates cancer advancement through activating the β-catenin-independent Wnt signaling pathway (Kagey et al. 2017). According to Chen et al., the β-catenin/MMP7 signaling pathway is responsible for the promotion of cell migration and invasion in HCC, which is facilitated by the high expression of DKK1 (Chen et al. 2013). DKK1 induces the expression of proteins related to cancer stem-like cells, promoting cell proliferation, migration, invasion, and VM in NSCLC (Yao et al. 2016). Furthermore, studies have revealed that elevated levels of DKK1 are linked to the poor prognosis of gliomas. Knockdown of DKK1 significantly reduced cell proliferation by inhibiting the PI3K-AKT pathway in glioma (Li et al. 2022). However, an in-depth mechanism of the functional role of FOXD1 and DKK1 in glioma VM remains unknown.

In our study, we aimed to clarify the molecular mechanism of SUMOylation of RALY in human glioma cells. In addition, we profiled the expression of UBA2, RALY, FOXD1, and DKK1 and explored the probable interaction mechanism among these molecules in the regulation of vasculogenic mimicry (VM) in glioma. Our findings may contribute to develop novel strategy for glioma therapy.

## Materials and methods

### Quantitative real-time PCR (qRT-PCR)

QRT-PCR was used to detect RNA expression. TRIzol reagent was used to extract total RNA from tissues and cells. Table S1 contains the list of primers. For further information, please refer to the Supporting Information for additional details.

### Cell culture and transfections

The U251 and U373 cell lines derived from human glioma were acquired from the Cell Resource Center (Institute of Biological Sciences, Shanghai, China). NHAs, which are normal human astrocytes, were acquired from Shanghai Zeye Biotechnology Co., Ltd. The knockdown plasmids, plasmids for overexpression, plasmids with mutations, and their corresponding plasmids for negative control were synthesized in GenePharma (Shanghai, China), GeneChem (Shanghai, China), and JTS (Beijing, China). The effectiveness of transfection was evaluated using Western blotting or qRT-PCR. For further information, please refer to the Supporting Information for additional details.

### Human tissue samples

Specimens of tissue samples were obtained from the Department of Neurosurgery of Shengjing Hospital affiliated to China Medical University. The Ethics Committee of Shengjing Hospital granted approval for this study, and all patients voluntarily provided their informed consent. For further information, please refer to the Supporting Information for additional details.

### Western blot

The protein expression was detected by Western blot. For further information, please refer to the Supporting Information for additional details.

### Migration and invasion assay

The transwell assay was used to detect the migratory and invasive capabilities of glioma cells. For further information, please refer to the Supporting Information for additional details.

### VM assays

The occurrence of VM in glioma cells was detected by the three-dimensional cell culture assay. To observe and capture images of the cellular vascular structure, an inverted microscope (Olympus, Tokyo, Japan) was employed. For further information, please refer to the Supporting Information for additional details.

### SUMOylation assays

The SUMOylation assay, as previously described by Deng et al. (2015) and Huang et al. (2012), identified RALY SUMOylation in the human glioma cell lines U251 and U373 using Ni2 + -nitrilotriacetic acid (NTA) beads.

### Immunofluorescent staining

Protein expression and subcellular localization were analyzed using immunofluorescence staining. For further information, please refer to the Supporting Information for additional details.

### RNA immunoprecipitation (RIP) assay

To identify the RNAs that are bound to RALY, a RIP assay was conducted. For further information, please refer to the Supporting Information.

### RNA stability measurement

At various time intervals, we extracted total RNA and measured the expression of FOXD1 mRNA using qRT-PCR. For further information, please refer to the Supporting Information.

### Protein half-life assay

After separating the cell extracts at each time point, they were subsequently analyzed using Western blotting. The time interval at which the concentration of RALY reaches 50% of the concentration at 0 h is known as its half-life. For more details, refer to the Supporting Information.

### Chromatin immunoprecipitation (ChIP) assay

To perform the ChIP assay, the ChIP Enzymatic Chromatin IP Kit was utilized. Specific primers were used to amplify the immunoprecipitated DNA through qRT-PCR. Table S2 provides a comprehensive list of the primer specifications. For additional information, please refer to the Supporting Information.

### CD34-PAS dual-staining

CD34-PAS was used to quantitatively and qualitatively analyze the VM in the xenograft tissue sections of nude mice. For additional information, please refer to the Supporting Information.

### Tumor xenografts in nude mice

Nude mice were used to establish xenograft models by introducing U251 and U373 glioma cells through stable transfection. For additional information, please refer to the Supporting Information.

### Statistical analysis

Experimental data is presented as the average plus or minus the standard deviation (SD). The statistical software SPSS 18.0 was used to conduct statistical analysis, which involved applying Student’s *t*-test (two-tailed) or one-way analysis of variance. The Kaplan–Meier method and log-rank test were utilized to conduct survival analysis.

## Results

### UBA2 promoted SUMOylation of RALY in cells

**Fig. 1:**
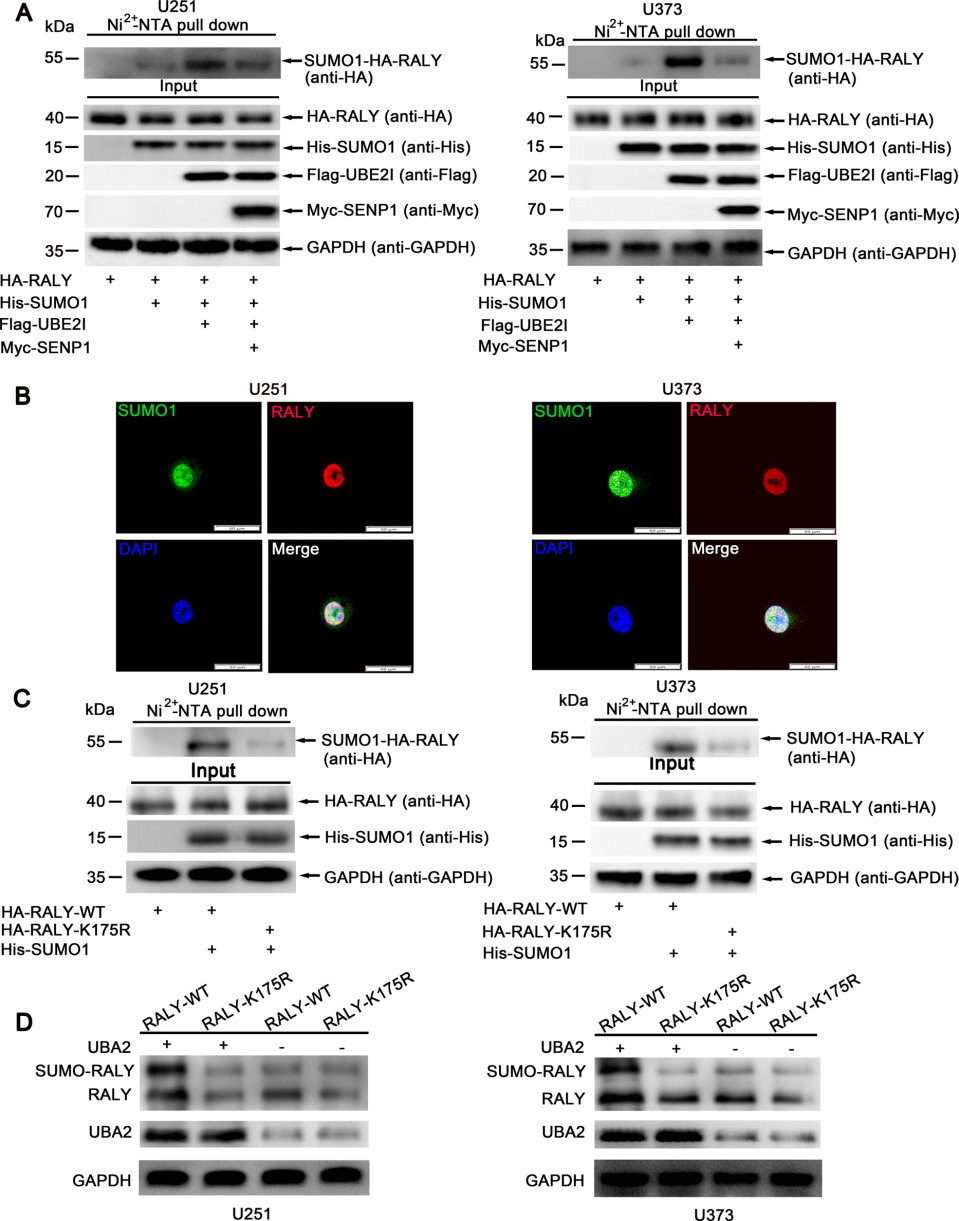
UBA2 promoted SUMOylation of RALY in cells. **A** RALY is modified by SUMO1 in glioma cells. U251 and U373 cells transfected with indicated plasmids were lysed and pulled down with Ni^2+^-NTA resin for SUMOylation assay, and SUMO1 modification of RALY was analyzed by Western blotting with indicated antibodies. **B** Laser scanning confocal microscopy observed the subcellular localization of RALY and SUMO1 in U251 cells and U373 cells. Scale bars: 50 µm. **C** K175R mutation weakens SUMO1 modification of RALY in U251 and U373 cells. The construct HA-RALY-WT or HA-RALY-K175R was co-transfected with His-SUMO1 into U251 and U373 cells. Cells were lysed for the SUMOylation assay with Ni^2+^-NTA resin. **D** Western blot was used to evaluate that UBA2 promotes the expression of RALY by SUMOylation

To validate our prediction that RALY could be SUMOylated, we first examined whether RALY could indeed be modified by SUMO1. The His-SUMO1, HA-RALY, Flag-UBE2I, and Myc-SENP1 plasmids were co-transfected into U251 and U373 cells to detect whether RALY could be SUMO1-modified in cells (Fig. 1A). The SUMOylation assay was conducted using the Ni^2+^-NTA resin pull-down method. The His-tagged SUMO1 conjugates were purified with Ni^2+^-NTA agarose beads as described in previous studies (Qu et al. 2014). The results showed that RALY could be SUMOylated by conjugation with SUMO1. The SUMO1 modification of RALY was facilitated by co-transfection with UBE2I but was abated by the addition of SENP1. An immunofluorescence analysis suggested that the RALY–SUMO1 complex is localized predominantly in the cell nucleus (Fig. 1B). We used the GPS-SUMO prediction tool (available at http://sumosp.biocuckoo.org.) to predict and score the potential SUMOylation sites in RALY and found that RALY contains only one lysine residue in the consensus motif ψKXD/E or E/DXKψ for SUMOylation (Figure S1A). Thereafter, using site-directed mutagenesis, we created the lysine 175 arginine (K175R) mutant of RALY. The wild-type (HA-RALY-WT) or mutant RALY (HA-RALY-K175R) and His-SUMO1 plasmids were co-transfected for the Ni^2+^-NTA resin pull-down assay. Compared with HA-RALY-WT, HA-RALY-K175R obviously reduced the SUMOylation level of RALY (Fig. 1C). Since UBA2 is a SUMO-activating enzyme subunit (Wang et al. 2020), we questioned whether UBA2 played a crucial role in the SUMOylation of RALY. To explore the function of UBA2, we co-transfected UBA2 with HA-RALY-WT or HA-RALY-K175R into U251 and U373 cells. The results demonstrated that UBA2 promoted the SUMOylation of RALY (Fig. 1D). In addition, the proteasome inhibitor MG132 could eliminate the degradation of RALY (Figure S1B). Furthermore, to examine the effect of UBA2 on the stability of RALY, we determined the half-life of RALY in U251 cells treated with inhibition of UBA2. As the results indicated, the half-life of RALY was significantly decreased from more than 16 h in the control group to less than 7 h in sh-UBA2 cells treated with actinomycin D (Figure S1C). The findings suggested that UBA2 promoted RALY SUMOylation, and K175 is a major SUMOylated amino acid residue on RALY.

### UBA2 expression was upregulated in glioma cells and UBA2 silencing suppressed cell migration, invasion, and VM

**Fig. 2:**
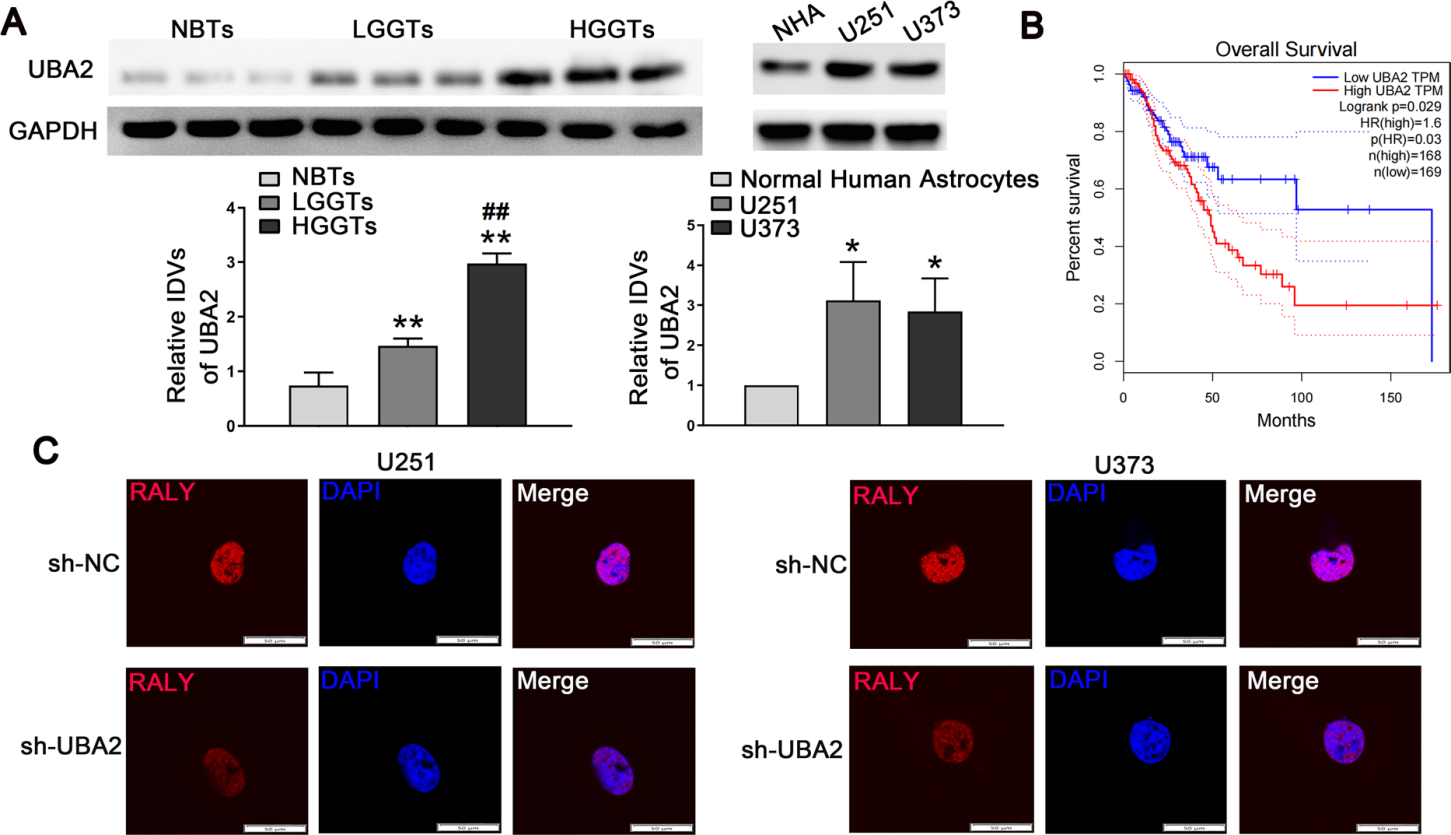
UBA2 expression was upregulated in glioma tissues and cells. **A** UBA2 protein expression levels in nontumorous brain tissues (NBTs) and glioma tissues using GAPDH as an endogenous control (left). Representative protein expression and their integrated density values (IDVs) of UBA2 in NBTs, low-grade glioma tissues (LGGTs: WHO I-II), and high-grade glioma (HGGTs: WHO III-IV) are shown. Data are presented as the mean ± SD (*n* = 3 in each group). **P < 0.01 versus NBTs group; ^*##*^*P* < 0.01 versus low-grade glioma tissues group. RALY protein expression levels in NHAs, U251, and U373 cells using GAPDH as an endogenous control (right). Representative protein expressions and their IDVs in NHA, U251, and U373 are shown. Data are presented as the mean ± SD (*n* = 3 in each group). *P < 0.05 versus NHA group. **B** Analysis of the overall survival of UBA2 in the data of glioma tissue in database (*n* (high) = 168, *n* (low) = 169), *P* < 0.05. **C** Laser scanning confocal microscope was used to observe the subcellular distribution of RALY protein in the UBA2 knockdown cells. Scale bars: 50 µm*.* Using one-way analysis of variance for statistical analysis

We detected the expression of UBA2 in glioma tissues and cells by Western blot. Compared with non-tumorous brain tissues (NBTs), glioma tissues exhibited an increased UBA2 expression, with U251 and U373 glioma cells showing higher levels than normal HA cells (Fig. 2A). The GEPIA database (http://gepia.cancer-pku.cn) showed that UBA2 expression was negatively correlated with the survival period in glioma patients (Fig. 2B). In addition, the immunofluorescence analysis suggested that UBA2 inhibition decreased the expression of RALY (Fig. 2C)*.* To investigate the function of UBA2, we created stable cell lines with UBA2 silenced. The cells in the sh-UBA2 group showed weak migration and invasion abilities (Figure S2A). Moreover, UBA2 inhibition significantly inhibited VM in glioma cells (Figure S2B). These results suggested that UBA2 inhibition suppressed the malignant progression in glioma cells.

### UBA2 exerted an oncogenic role by regulating RALY expression in glioma cells

**Fig. 3:**
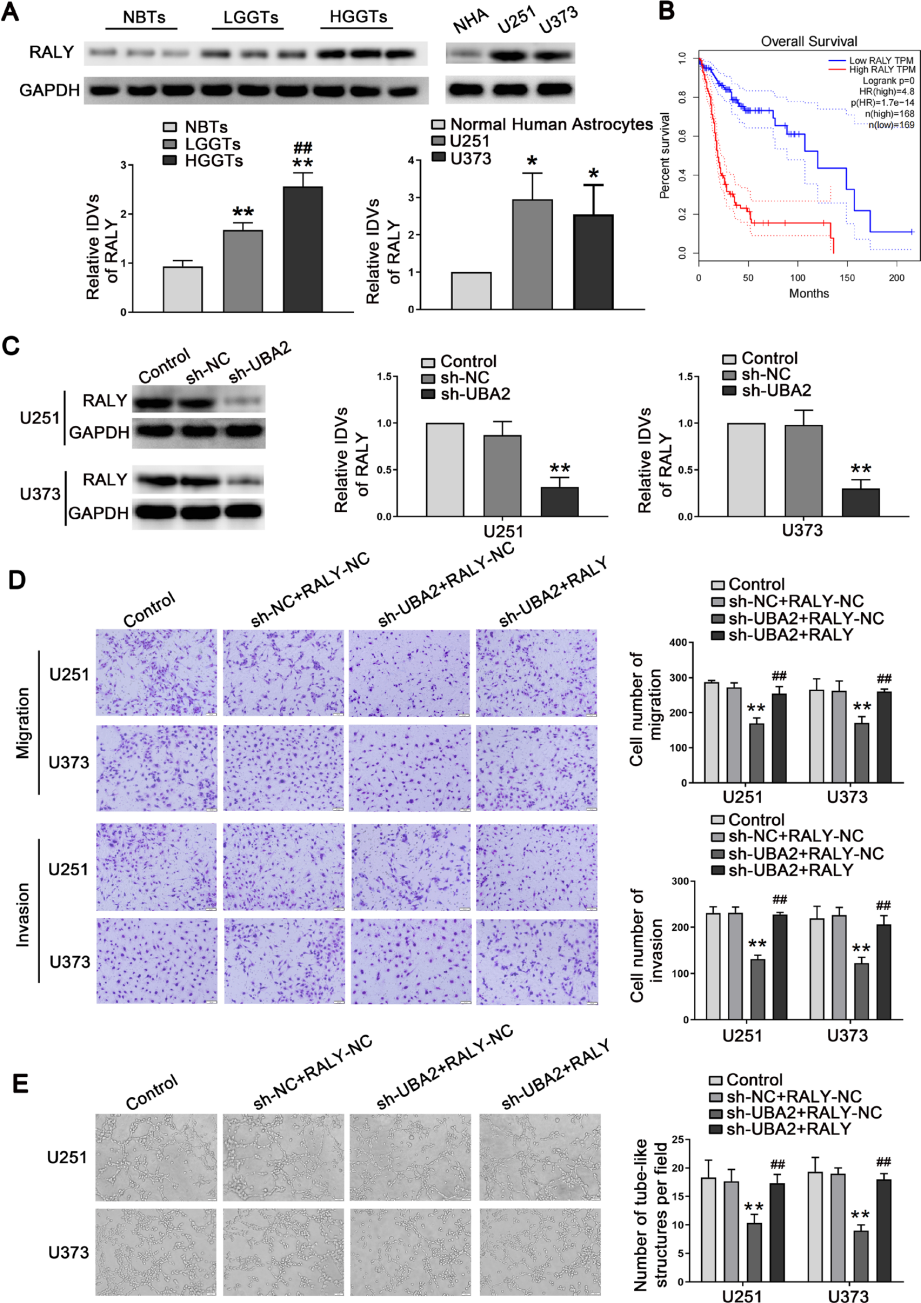
UBA2 regulates the abilities of migration, invasion, and VM formation in glioma cells by regulating the expression of RALY. **A** Western blot was used to detect the expression of RALY protein in NBTs, LGGTs, and HGGTs using GAPDH as an endogenous control (left). Data are presented as the mean ± SD (*n* = 3). **P < 0.01 versus NBTs group; ^*##*^*P* < 0.01 versus LGGs group. RALY protein expression levels in NHA, U251, and U373 cells using GAPDH as an endogenous control (right). Data are presented as the mean ± SD (*n* = 3 in each group). *P < 0.05 versus NHA group. **B** Analysis of the overall survival of RALY in the data of glioma tissue in database (*n* (high) = 168, *n* (low) = 169), *P* < 0.05. **C** Western blot assay was used to detect the expression of RALY in cells treated with inhibition of UBA2. Data are presented as the mean ± SD (*n* = 3 in each group). **P < 0.01 versus sh-NC group (empty vectors). **D** Quantification number of migration and invasion cells treated with altered expression of UBA2 and RALY. **E** Three-dimensional cell culture method was used to detect the change of VM in the cells treated with altered expression of UBA2 and RALY on U251 and U373 cells. Representative images and accompanying statistical plots were presented. Data are presented as the mean ± SD (*n* = 3 in each group). **P < 0.01 versus sh-NC + RALY-NC group; ^*##*^*P* < 0.01 versus sh-UBA2 + RALY-NC group; Scale bars represent 50 µm. Using one-way analysis of variance for statistical analysis

RALY expression was markedly increased in glioma (Fig. 3A). According to the GEPIA database, high RALY expression leads to poor overall survival (Fig. 3B). Functional experiments were conducted to explore the effect of RALY. The results indicated that overexpression of RALY facilitated cell migration, invasion, and VM, whereas its knockdown suppressed cell migration, invasion, and VM (Figure S3A, B). Next, we explored the effect of UBA2 on RALY expression, which revealed that RALY was significantly decreased in the UBA2 inhibition group (Fig. 3C). Furthermore, transwell and VM assay results demonstrated that overexpression of RALY rescued the effect of UBA2 inhibition on cell migration, invasion, and VM (Fig. 3D, E). These results demonstrated that UBA2 facilitated the malignant advancement through regulating of RALY expression in glioma cells.

### RALY plays an oncogenic role by stabilizing FOXD1

**Fig. 4:**
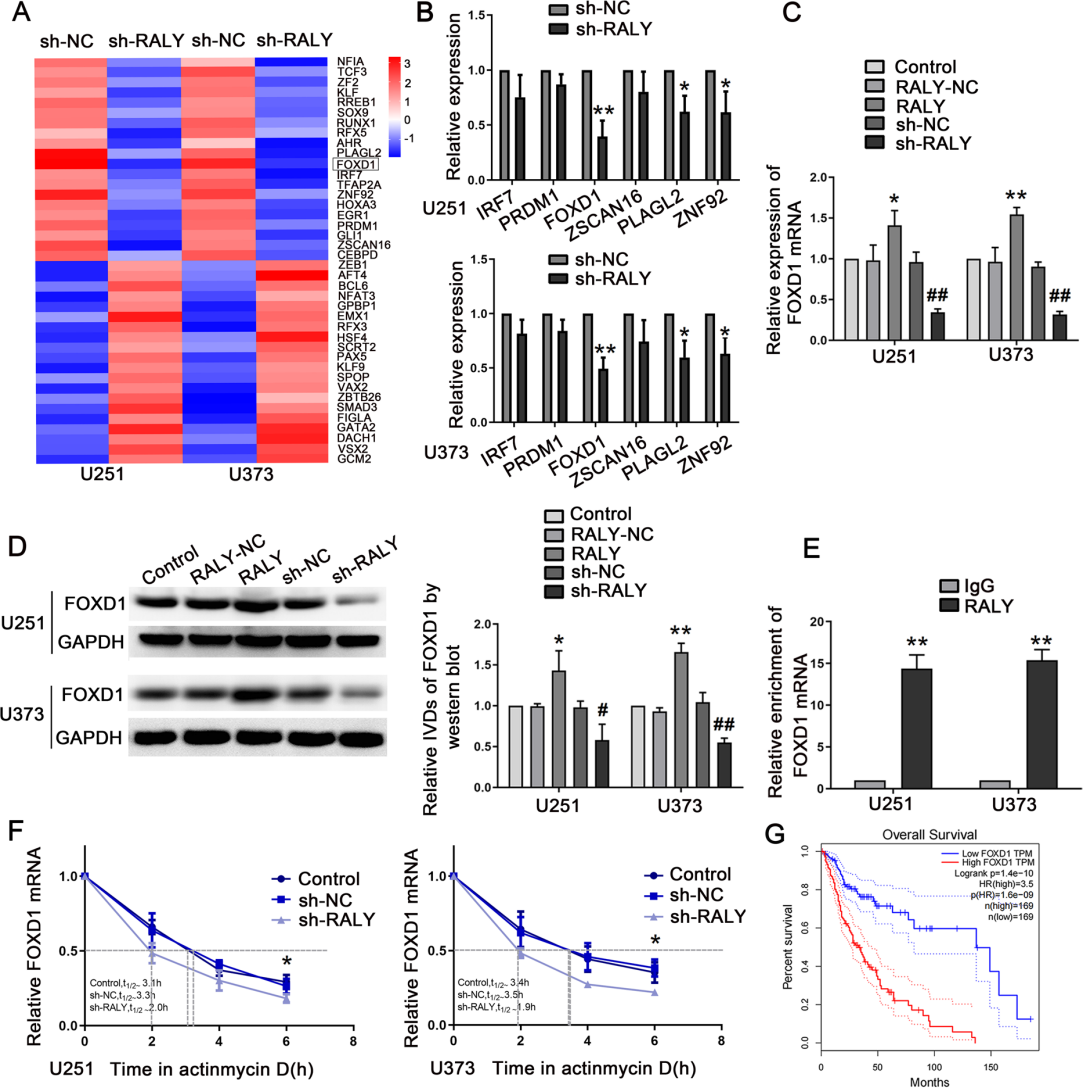
RALY promoted VM formation in glioma cells via stabilizing FOXD1. **A** Microarray assay was performed to detect the differential gene expression profiles when RALY was knockdown. **B** qRT-PCR was performed to validate the selected molecules. Data are presented as the mean ± SD (*n* = 3 in each group). *P < 0.05, **P < 0.01 versus sh-NC group. Using Student’s *t*-test for statistical analysis. **C** qRT-PCR was used to detect the expression of FOXD1 mRNA in the cells treated with altered expression of RALY. Each value represents the mean ± SD (*n* = 3). *P < 0.05, **P < 0.01 versus RALY-NC group; ^*##*^*P* < 0.01 versus sh-NC group. Using one-way analysis of variance for statistical analysis. **D** Western blot was used to detect the expression of FOXD1 protein in the cells treated with altered expression of RALY. Representative protein expressions and corresponding IDVs of FOXD1 in U251 and U373 are shown; data are presented as mean ± SD (*n* = 3, each group). *P < 0.05, **P < 0.01 versus RALY-NC group; ^*#*^*P* < 0.05, ^*##*^*P* < 0.01 versus sh-NC group. Using one-way analysis of variance for statistical analysis. **E** FOXD1 mRNA was identified in the RALY complex. Relative enrichment of FOXD1 mRNA was measured using qRT-PCR. Data represent mean ± SD (*n* = 3 in each group). **P < 0.01 versus anti-normal IgG group. Using Student’s *t*-test for statistical analysis. **F** The graph represents the relative levels of the FOXD1 mRNA at the different actinomycin D treatment times in the control group, sh-NC group, and sh-RALY group (*n* = 5 in each group of U251 cells, *n* = 3 in each group of U373 cells). *P < 0.05 versus sh-NC group. Using one-way analysis of variance for statistical analysis. **G** Analysis of the overall survival of FOXD1 in the data of glioma tissue in database (*n* (high) = 169, *n* (low) = 169), *P* < 0.001

A microarray analysis showed that RALY inhibition caused the downregulation of several transcription factors in U251 and U373 cells. Of these, six most relevant transcription factors were selected by cross-screening with the transcription factors available in the JASPAR database (http://jaspar.genereg.net/). The expression of FOXD1 in the sh-RALY group exhibited a more significantly decrease compared to the sh-NC group (Fig. 4A, B). As shown in Fig. 4C, D, the results indicated that the overexpression of RALY upregulated *FOXD1* mRNA and protein expression. In addition, overexpression of RALY rescued the decreased expression of FOXD1 due to UBA2 knockdown (Figure S4A, B). Next, we examined the correlation between *RALY* and *FOXD1* mRNAs. According to starBase software (http://starbase.sysu.edu.cn/), we predicted that RALY may bind to *FOXD1* mRNA. Furthermore, the direct binding of RALY to *FOXD1* mRNA was detected via an RNA immunoprecipitation (RIP) assay. Compared to the IgG group, *FOXD1* mRNA enrichment in the RALY group was significantly higher (Fig. 4E). Subsequently, we examined the half-life of *FOXD1* mRNA after knocking-down RALY, and the results showed a significant reduction in the half-life of FOXD1 in sh-RALY cells (Fig. 4F). According to the GEPIA database, high FOXD1 expression leads to poor overall survival (Fig. 4G). Furthermore, to explore the effect of FOXD1 on cell-driven angiogenesis, we created FOXD1-overexpression and -silenced stable cell lines. Glioma cells treated with FOXD1 inhibition showed decreased VM, whereas those overexpressing FOXD1 showed enhanced VM (Figure S4C). Moreover, the overexpression of FOXD1 rescued the suppression of migration, invasion, and VM induced by RALY knockdown in glioma cells (Figure S5A, S5B). The above results indicated that the UBA2/RALY axis regulated the expression of FOXD1 by increasing its stability.

### DKK1 was upregulated in glioma cells and DKK1 knockdown suppressed glioma cell migration, invasion, and VM

**Fig. 5:**
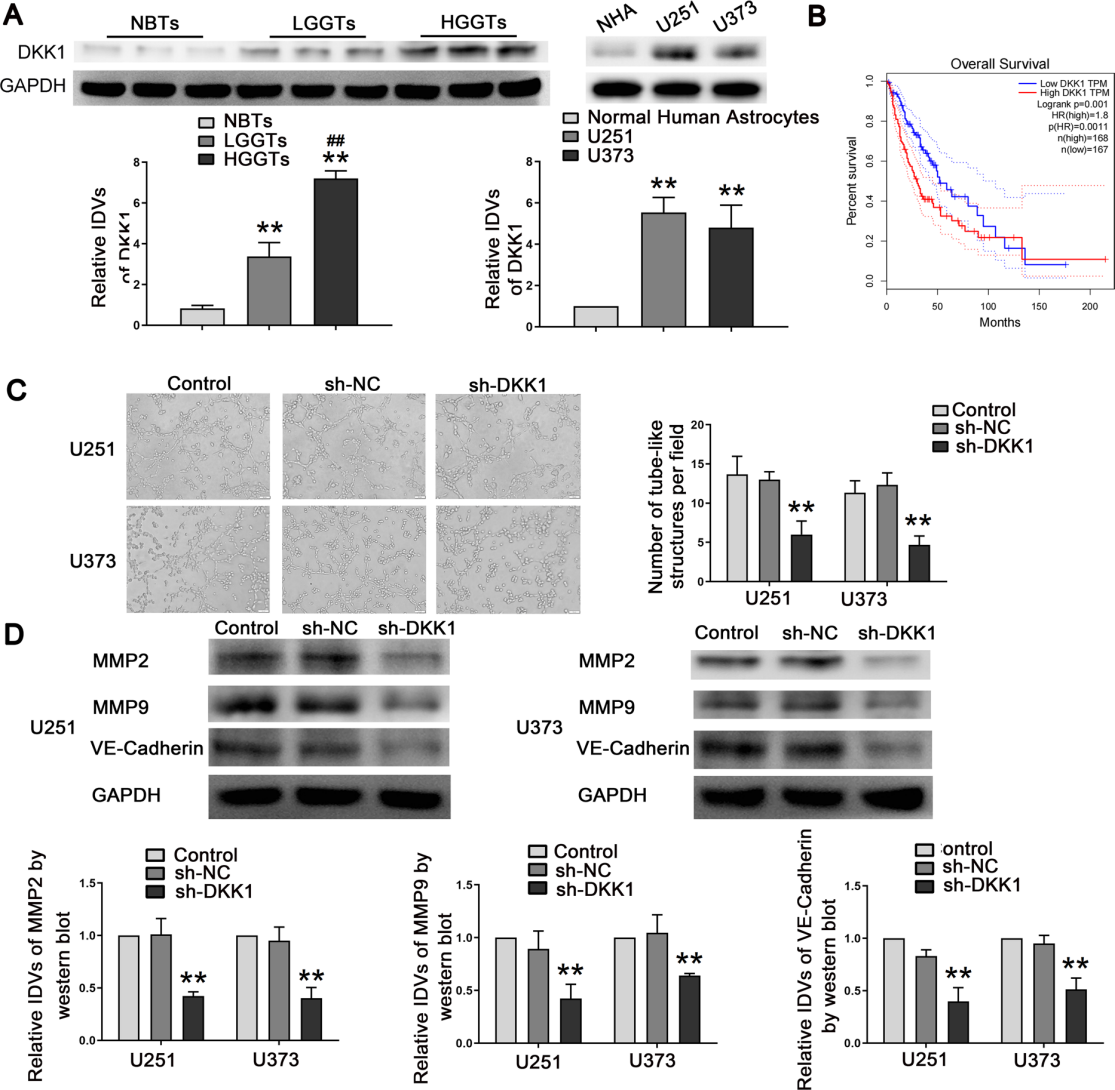
DKK1 was up-regulated in glioma tissues and cells, and knockdown of DKK1 inhibited migration, invasion and VM formation. **A** Western blot was used to detect the expression of DKK1 protein in NBTs, LGGTs, and HGGTs (left). Data are presented as the mean ± SD (*n* = 3). **P < 0.01 versus NBTs group; ^*##*^*P* < 0.01 versus LGGTs group. DKK1 protein expression levels in NHA, U251, and U373 cells (right). Data are presented as the mean ± SD (*n* = 3 in each group). **P < 0.01 versus NHA group. **B** Analysis of the overall survival of DKK1 in the data of glioma tissue in database (*n* (high) = 168, *n* (low) = 167), *P* < 0.05. **C** Three-dimensional cell culture method was used to detect the change of VM in the cells treated with inhibition of DKK1 on U251 and U373 cells. Representative images and accompanying statistical plots were presented. Data are presented as the mean ± SD (*n* = 3 in each group). **P < 0.01 versus sh-NC group; Scale bars represent 50 µm. **D** Western blot assay was used to detect the expression of MMP2, MMP9, and VE-Cadherin in cells treated with inhibition of DKK1. Data are presented as the mean ± SD (*n* = 3 in each group). **P < 0.01 versus sh-NC group (empty vectors). Using one-way analysis of variance for statistical analysis

A recent study has confirmed that DKK1 overexpression promotes VM in NSCLC tumor cells. Here, we investigated the functional role of DKK1 on glioma cells. It was observed that the DKK1 expression was significantly elevated in glioma (Fig. 5A). According to the GEPIA database, for patients with glioma, higher DKK1 expression is associated with shorter survival time (Fig. 5B). Subsequently, we explored the effects of DKK1 knockdown and found that it attenuated the vasculogenic networks in glioma cells and decreased the expression levels of metalloproteinases MMP2 and MMP9 and VE-Cadherin (Fig. 5C, D). In addition, DKK1 knockdown significantly inhibited cell migration and invasion (Figure S6A). These results suggested that DKK1 has a carcinogenic effect on glioma cells.

### FOXD1 promoted glioma cell malignant progression by binding to the DKK1 promoter

**Fig. 6:**
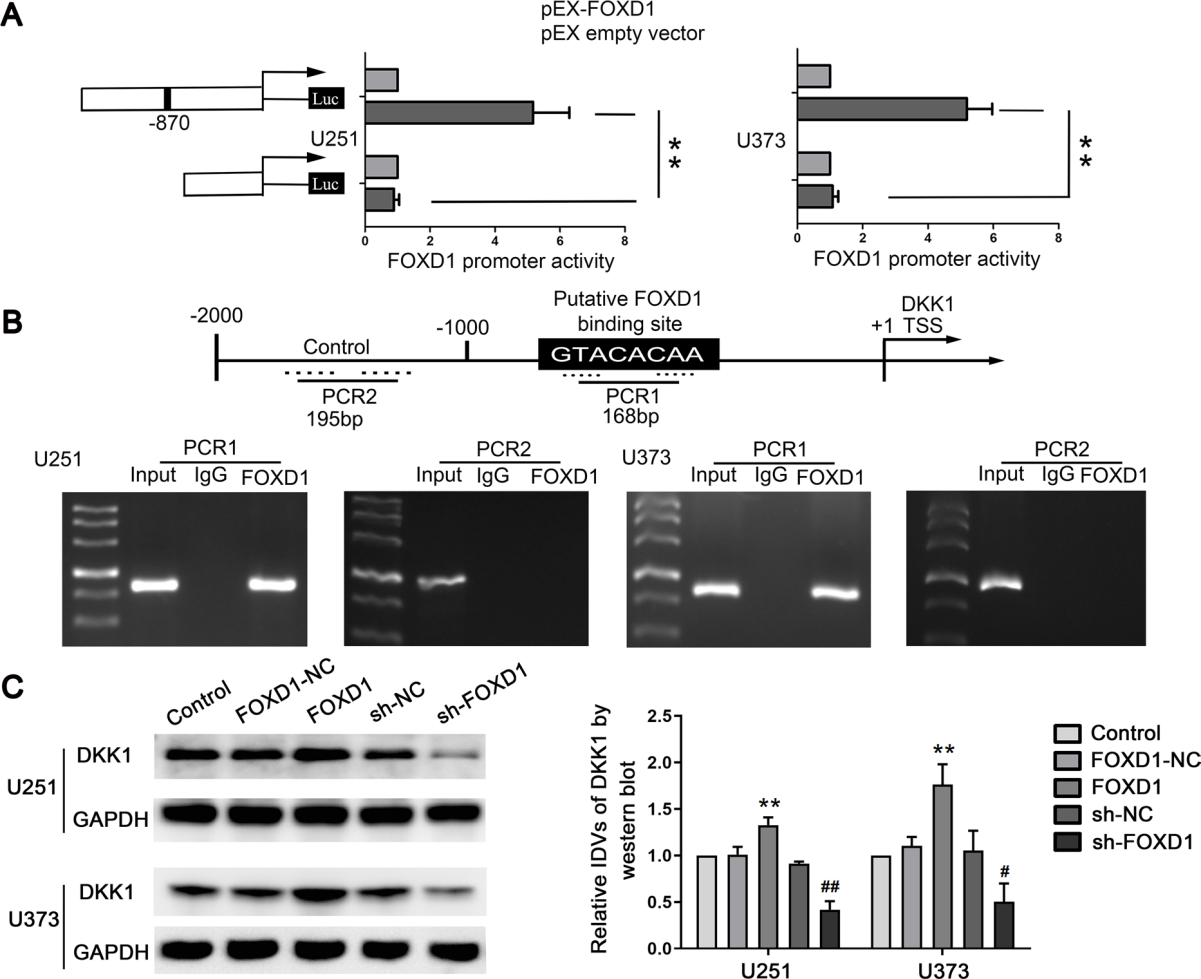
FOXD1 directly binds to the promoter and upregulates the expression of DKK1. **A** The effect of FOXD1 on DKK1 promoter activity. Data represents mean ± SD (*n* = 3, each). **P < 0.01. **B** Putative FOXD1 binding sites are shown. Immunoprecipitated DNA was amplified by PCR. Normal rabbit IgG was used as a negative control. **C** Western blot analysis for FOXD1 regulating DKK1 expression in U251 and U373 cells. **P < 0.01 versus FOX1-NC group; ^#^*P* < 0.05, ^##^*P* < 0.01 versus sh-NC group. Using one-way analysis of variance for statistical analysis

A luciferase assay was performed to explore whether FOXD1 directly bound to the DKK1 promoter. The removal of the presumed binding site (− 870 bp) significantly decreased the promoter activity of FOXD1 (Fig. 6A). Moreover, the chromatin immunoprecipitation (ChIP) assays indicated a direct interaction between FOXD1 and DKK1 but no association between FOXD1 and the control region (Fig. 6B). Furthermore, the expression of DKK1 was investigated in U251 and U373 cells that were treated with FOXD1 or sh-FOXD1. The overexpression of FOXD1 significantly increased the DKK1 protein expression (Fig. 6C). In the rescue experiment, the decrease in DKK1 expression levels caused by inhibiting RALY was elevated by the overexpression of FOXD1 (Figure S7A). Moreover, overexpression of DKK1 rescued the inhibition of migration, invasion, and VM induced by FOXD1 knockdown (Figure S7B, C). These results confirmed that FOXD1 promoted glioma cell migration, invasion, and VM by promoting DKK1 expression. The results confirmed that FOXD1 transcriptionally activated DKK1 expression in glioma cells.

### Combined inhibition of UBA2, RALY, and FOXD1 restrained tumor growth and prolonged survival time in nude mice

Based on the above findings, stable knockdowns of UBA2, RALY, and FOXD1 alone or in combination were constructed to investigate their inhibitory effects on glioma cells. The combined inhibition of UBA2, RALY, and FOXD1 significantly impeded glioma cell migration, invasion, and VM compared with the control group. Moreover, the sh-UBA2 + sh-RALY + sh-FOXD1 group had the highest inhibitory effect among all groups (Figure S8A, B).

**Fig. 7:**
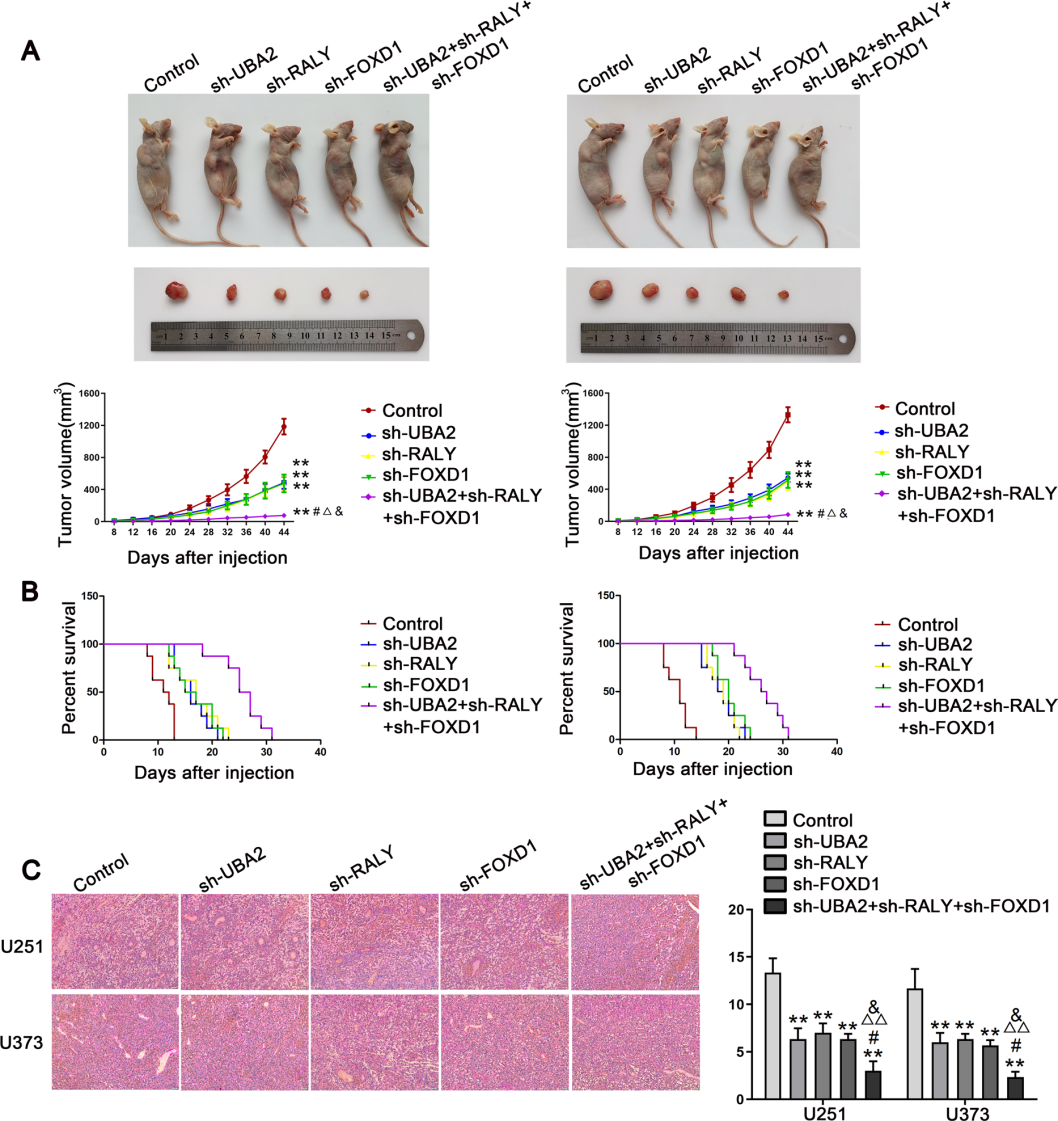
Tumor xenograft studies. **A** The stable expressing cells were used for the in vivo study. The nude mice carrying tumors from respective groups are shown. The sample tumors from respective groups are shown. Tumor volume was calculated every 4 days after injection, and the tumor was excised after 44 days. Each value represents the mean ± SD (*n* = 3), **P < 0.01 versus control group; ^#^*P* < 0.05, ^##^*P* < 0.01 versus sh-UBA2 group; ^△^*P* < 0.05 versus sh-RALY group; ^&^*P* < 0.05 versus sh-FOXD1 group. Using one-way analysis of variance for statistical analysis. **B** The survival curves of nude mice with xenografts injected into the right striatum. *P* < 0.05 for sh-UBA2, sh-RALY, or sh-FOXD1 versus control group; *P* < 0.01 for sh-UBA2 + sh-RALY + sh-FOXD1 versus control group; *P* < 0.05 for sh-UBA2 + sh-RALY + sh-FOXD1 versus sh-UBA2, sh-RALY, or sh-FOXD1 group. Using a log-rank test for statistical analysis. **C** CD34-PAS staining was used to detect the effects of sh-UBA2, sh-RALY, and sh-FOXD1 on VM in orthotopic transplanted tumor tissue of nude mice, alone or in combination. Scale bars: 50 µm. Each value represents the mean ± SD (*n* = 8), **P < 0.01 versus control group; ^#^*P* < 0.05 versus sh-UBA2 group; ^△△^*P* < 0.01 versus sh-RALY group; ^&^*P* < 0.05 versus sh-FOXD1 group. Using one-way analysis of variance for statistical analysis

Subcutaneous and orthotopic xenograft models were constructed in nude mice to detect the functions of the UBA2/RALY/FOXD1 axis in vivo. Compared with the control groups, the sh-UBA2, sh-RALY, and sh-FOXD1 groups showed smaller tumor volumes. Moreover, the combined knockdown of UBA2, RALY, and FOXD1 resulted in the smallest tumor volume (Fig. 7A). Furthermore, the analysis of survival revealed that the groups with sh-UBA2, sh-RALY, and sh-FOXD1 exhibited extended survival time. Moreover, the sh-UBA2 + sh-RALY + sh-FOXD1 group had the longest survival period (Fig. 7B). Finally, the CD34-PAS staining was performed on the pathological sections of orthotopically transplanted tumors in nude mice to detect the density of VM in each group. The results showed that the sh-UBA2, sh-RALY, and sh-FOXD1 groups had lower VM density than the control groups. Meanwhile, the sh-UBA2 + sh-RALY + sh-FOXD1 group had the lowest VM density (Fig. 7C).

## Discussion

**Fig. 8:**
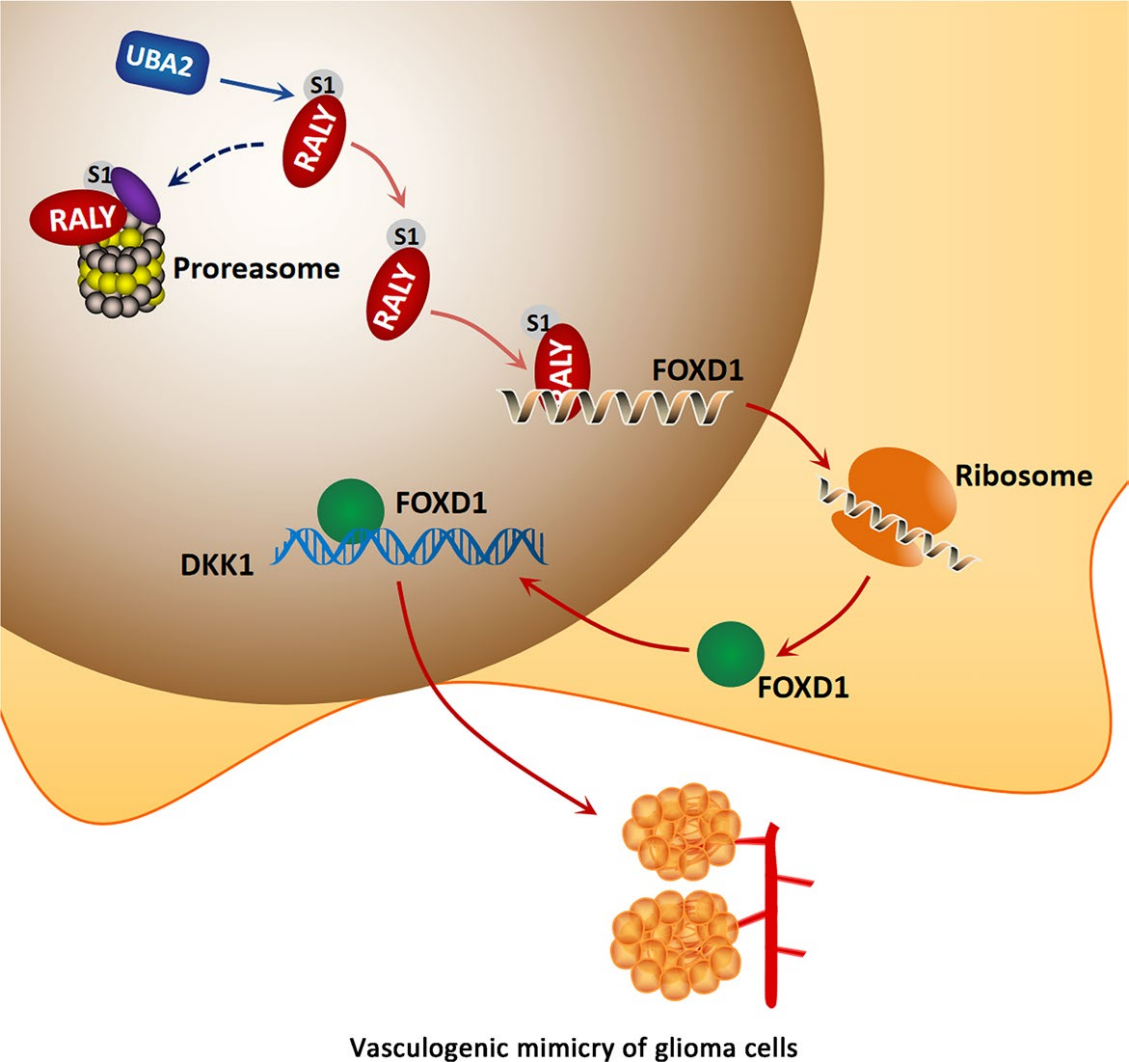
The schematic cartoon of the mechanism of UBA2 promoted SUMOylation of RALY to regulate vasculogenic mimicry of glioma cells. The expression of UBA2 is significantly increased, thereby promoting RALY SUMOylation, inhibiting the degradation of RALY protein by proteasome, and increasing the stability of RALY to upregulate the expression of RALY. RALY upregulates FOXD1 mRNA and protein expression via increasing its stability. FOXD1 overexpression promotes the transcriptional expression of DKK1 and then promotes the migration, invasion, and VM of glioma cells

This study demonstrated that RALY was modified by SUMO1 at the major conjugation site K175 in glioma cells. The increased UBA2 expression in glioma cells promoted RALY SUMOylation, which, in turn, upregulated the expression of RALY by increasing its stability. Inhibition of RALY hindered migration, invasion, and VM in glioma cells via FOXD1 destabilization. Meanwhile, FOXD1 enhanced the activity of DKK1 promoter by directly interacting with it. Moreover, DKK1 knockdown inhibited glioma cell migration, invasion, and VM (Fig. 8).

UBA2 is the sole E1-activating enzyme required for the SUMOylation of many important proteins. The abnormal expression of UBA2 is associated with carcinogenesis (Lv et al. 2018). In HCC, UBA2 is highly expression and promotes cell proliferation and colony formation by facilitating the SUMOylation of the multifunctional transcription factor TFII-I (Tu et al. 2015). In renal cell carcinoma, significantly increased UBA2 expression is related to higher stages and poorer prognosis. Downregulation of UBA2 promoted apoptosis and inhibited proliferation (Zhang et al. 2021). In small cell lung cells, UBA2 expression is significantly upregulated; however, its knockdown suppresses the migration, invasion, and growth of lung cells and increases their sensitivity to chemotherapeutic drugs cisplatin and etoposide (Liu et al. 2015). Similar to the results of these studies, UBA2 demonstrated oncogenic activity in glioma cells. In this study, we discovered that UBA2 demonstrated elevated expression levels in glioma. Knockdown of UBA2 impeded the malignant progression of glioma cells.

SUMOylation is a reversible post-translational modification, which plays an important regulatory role in various physiological processes such as transcriptional activation, subcellular localization, protein–protein interaction, and protein stability (Son et al. 2023). SUMO1 is upregulated in glioma cells and tissues, and the SUMO1 modification of the cell cycle regulator CDK6 could upregulate its expression by stabilizing it and, thus, could promote cell malignant progression (Bellail et al. 2014). In gastric cancer cells, the AKT/mTOR signaling pathway was hindered by TUFT1 SUMOylation, leading to promotion of cell proliferation and migration (Wang et al. 2023). Moreover, reversible SUMO1 modification of hnRNP-K inhibits its ubiquitination for stabilization, thus enhancing its function as a p53 transcriptional co-activator, which can upregulate the expression of p53 and p21 (Lee et al. 2012). In addition, SUMOylation of hnRNP A2/B1 can promote the exosome-sorting process of miR-204-3p to expedite the progression of accelerate tumor growth and angiogenesis in glioma cells (Guo et al. 2023).

Similarly, in our study, the immunofluorescence analysis showed that RALY and SUMO1 are mainly colocalized in the nucleus of glioma cells. We predicted that the RALY sequence 174IKTE177 constitutes a typical ΨKxD/E SUMOylation motif, and the lysine residue can be modified by the SUMO protein. Furthermore, the Ni^2+^-NTA resin pull-down assay demonstrated that RALY can be modified by SUMO1 at amino acid residue K175, and the addition of the deSUMOylation enzyme SENP1 could weaken this modification. Moreover, UBA2 promoted the SUMOylation of RALY, which, in turn, increased its stability and upregulated the expression of RALY. The GEPIA database predicted a positive correlation between UBA2 expression and survival time in patients with glioma. Our data suggested that UBA2 is increased in gliomas, and its knockdown impedes cell migration, invasion, and VM. These results proved that UBA2 functions as an oncogene by promoting RALY SUMOylation in glioma cells.

According to the GEPIA database, glioma patients with high RALY expression were found to have a low overall survival rate. This study showed that RALY expression was upregulated, and UBA2 knockdown significantly downregulated RALY expression. Furthermore, the overexpression of RALY facilitated the migration, invasion, and vasculogenic mimicry of glioma cells and, more importantly, rescued the suppression of migration, invasion, and VM induced by UBA2 knockdown. These findings demonstrated that UBA2 facilitated the malignant progression in glioma cells by regulating the expression of RALY. Similar to the results of this study**,** in breast cancer cells, RALY expression is increased and negatively related to the patients’ survival period. Moreover, RALY could promote cell invasion by upregulating the expression of protein arginine methyltransferase isoform PRMT1v2 (Bondy-Chorney et al. 2017). In NSCLC, RALY expression is notably upregulated and associated with a higher incidence of lymph node metastasis and poorer patient survival. Silencing of RALY inhibits cell proliferation, migration, and invasion (Song et al. 2020).

In our study, the RIP assay demonstrated that RALY could bind to *FOXD1* mRNA. Besides, the inhibition of RALY downregulated the expression of *FOXD1* mRNA by decreasing its half-life. RALY upregulates *FOXD1* mRNA and protein expression by increasing its stability. Studies have shown that FOXD1 is upregulated and inversely related to patient survival in renal cell carcinoma (Bond et al. 2021). In oral squamous cell cancer, FOXD1 is highly expressed and predicts a poorer prognosis. The overexpression of FOXD1 promotes the chemoresistance and EMT of cells. Moreover, FOXD1 upregulates the expression of the lncRNA CYTOR by directly activating its promoter (Chen et al. 2021). In lung cancer cells, FOXD1 causes the transcriptional activation of Gal-3 by binding to its promoter, thus upregulating its expression and further promoting cell growth and motility. In accordance with its oncogenic role in these studies, the GEPIA database revealed a negative correlation between FOXD1 expression and the overall survival of patients with glioma. In addition, FOXD1 inhibition hindered cell migration, invasion, and vasculogenic mimicry. Furthermore, overexpression of FOXD1 rescued the suppression of migration, invasion, and VM induced by RALY knockdown in glioma cells. The above findings indicated that RALY regulated the expression of FOXD1 to promote a malignant progression of glioma cells.

FOXD1, a transcription factor, has been found to regulate the expression of genes downstream by directly binding to their promoters. In oral cancer cells, FOXD1 promotes the malignant progression of cells by upregulating *G3BP2* expression via directly binding to its promoter (Lin et al. 2020). As previously reported, DKK1 is upregulated in glioma cells and tissues, which is similar to the results of this study. Additionally, in an in *vivo* study, the overexpression of DKK1 in mouse glioma cells promotes tumor growth, angiogenesis, and subcutaneous xenograft tumor formation in nude mice (Reis et al. 2012). However, the effect of DKK1 on migration, invasion, and VM in glioma cells remains unclear. In this study, it showed the highly expression of DKK1 in glioma. Silencing DKK1 suppressed the migration, invasion, and vasculogenic mimicry of cells. Luciferase assay indicated that FOXD1 directly bound to the DKK1 promoter. Moreover, the chromatin immunoprecipitation (ChIP) assays indicated a direct interaction between FOXD1 and DKK1. Furthermore, overexpression of FOXD1 significantly increased the DKK1 protein expression. Overexpression of DKK1 rescued the suppression of migration, invasion, and VM induced by FOXD1 knockdown in glioma cells. These findings confirmed that FOXD1 transcriptionally activated DKK1 expression to exert oncogenic role in glioma cells.

In this study, we found that the expression levels of UBA2, RALY, FOXD1, and DKK1 were significantly increased in glioma cells and tissues and were negatively correlated with the overall survival time in glioma patients. The Ni^2+^-NTA resin pull-down assay confirmed that RALY can be SUMOylated by conjugation with SUMO1 at the major site K175. UBA2 promoted RALY SUMOylation and upregulated RALY protein expression by stabilizing it. Furthermore, the RIP results confirmed the existence of binding sites between RALY and *FOXD1* mRNA. Besides, our results demonstrated that RALY knockdown reduced *FOXD1* mRNA and protein expression by reducing the half-life of *FOXD1* mRNA. Moreover, we have confirmed that FOXD1 directly binds to the promoter of DKK1 and promotes its expression. In addition, we found that the inhibition of UBA2, RALY, FOXD1, and DKK1 inhibited glioma cell migration, invasion, and vasculogenic mimicry. Remarkably, the combined knockdown of UBA2, RALY, and FOXD1 largely suppressed xenograft tumor growth and VM and prolonged nude mice survival.

## Conclusions

This study revealed that the expression levels of UBA2 and RALY in glioma cells and tissues are significantly increased, and RALY can be modified by SUMO1 in glioma. The knockdown of UBA2 downregulated the expression of RALY by inhibiting its SUMOylation, thereby reducing its stability. RALY upregulates *FOXD1* mRNA and protein expression by increasing its stability. Silencing of FOXD1 downregulated the expression of DKK1 by inhibiting its transcription and, thus, decreased migration, invasion, and vasculogenic mimicry in glioma cells. Interactions among UBA2, RALY, FOXD1, and DKK1 play an important role in regulating migration, invasion, and vasculogenic mimicry in glioma cells. To our knowledge, this is the first study to highlight the role of UBA2 in regulating the migration, invasion, and VM of glioma cells via the RALY/FOXD1/DKK1 pathway, suggesting that blockade of the UBA2/RALY/FOXD1/DKK1 axis may be a potential treatment for gliomas. The inhibitor of UBA2, RALY, FOXD1, and DKK1 may serve as the therapeutic target in glioma. However, there still are several limitations, such as the development of brain penetrant inhibitors of UBA2, RALY, FOXD1, and DKK1 that can be selective against tumor cells as well as more studies for clinical research. Therefore, further clinical investigations of UBA2, RALY, FOXD1, and DKK1 inhibition monotherapy or combination therapy are necessary.

### Supplementary Information

Below is the link to the electronic supplementary material.

Supplementary file1 (DOC 1646 KB)

Supplementary file2 (DOC 3645 KB)

Supplementary file3 (DOC 2736 KB)

Supplementary file4 (DOC 4194 KB)

Supplementary file5 (DOC 3829 KB)

Supplementary file6 (DOC 2511 KB)

Supplementary file7 (DOC 5465 KB)

Supplementary file8 (DOC 3282 KB)

Supplementary file9 (DOCX 11 KB)

Supplementary file10 (DOCX 11 KB)

Supplementary file11 (DOC 51 KB)

## Data Availability

The data that support the findings of this study are available from the corresponding author upon reasonable request.

## Abbreviations

*NBTs*: Normal brain tissues
*NHAs*: Normal human astrocytes
*qRT-PCR*: Quantitative real-time PCR
*RIP*: RNA-binding protein immunoprecipitation
*ChIP*: Chromatin immunoprecipitation
*VM*: Vasculogenic mimicry
*NTA*: Ni^2+^-nitrilotriacetic acid
*EPC*: Endothelial progenitor cell
*NSCLC*: Non-small-cell lung cancer
*HCC*: Hepatocellular carcinoma
*SUMO*: Small ubiquitin-like modifier
*UBA2*: Ubiquitin-like modifier-activating enzyme 2
*hnRNP*: Heteronuclear ribonucleoprotein
*DKK1*: Dickkopf-1
*EMT*: Epithelial-to-mesenchymal transition
