# Supplementary material for: SUMOylation of RALY promotes vasculogenic mimicry in glioma cells via the FOXD1/DKK1 pathway
Source: Cell Biol Toxicol. 2023 Oct 31;39(6):3323–40. doi: 10.1007/s10565-023-09836-3 (PMC10693529; doi:10.1007/s10565-023-09836-3)
Supplement: Supplementary file 1 — Supplementary file1 (DOC 1646 KB) [file 10565_2023_9836_MOESM1_ESM.doc]

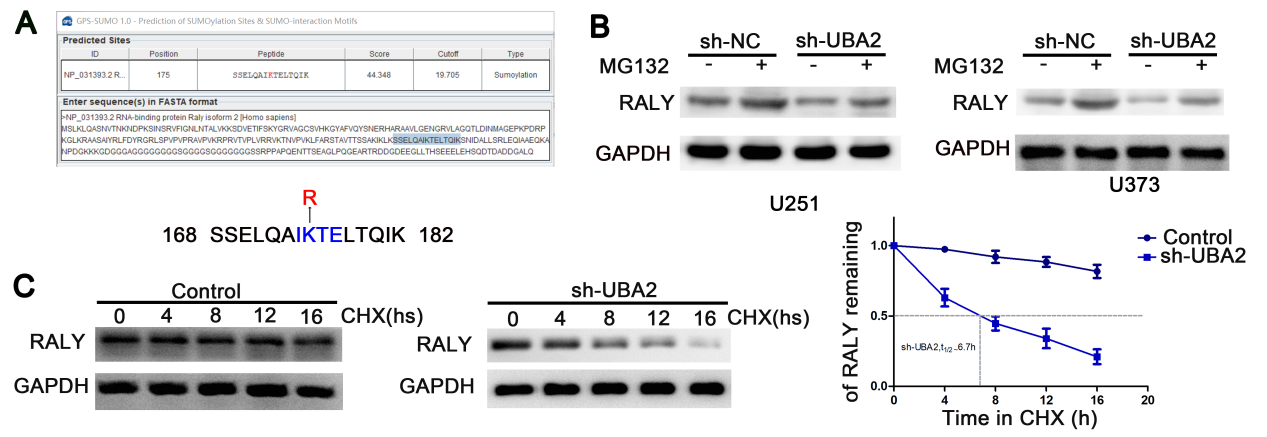
**Supplementary Figure 1**. (A) Potential SUMOylation sites in RALY. The Lys residues in the underlined sequences of RALY were substituted with Arg by site-directed mutagenesis. (B) Western blot was used to evaluate that MG132 inhibits the degradation of RALY promoted by SUMOylation. (C) The half-life of RALY protein was evaluated by western blotting. Data are presented as the mean±SD (n=3 in each group). ***P*<0.01 versus control group. Using Student’s t test for statistical analysis.
