# Supplementary material for: SUMOylation of RALY promotes vasculogenic mimicry in glioma cells via the FOXD1/DKK1 pathway
Source: Cell Biol Toxicol. 2023 Oct 31;39(6):3323–40. doi: 10.1007/s10565-023-09836-3 (PMC10693529; doi:10.1007/s10565-023-09836-3)
Supplement: Supplementary file 2 — Supplementary file2 (DOC 3645 KB) [file 10565_2023_9836_MOESM2_ESM.doc]

**
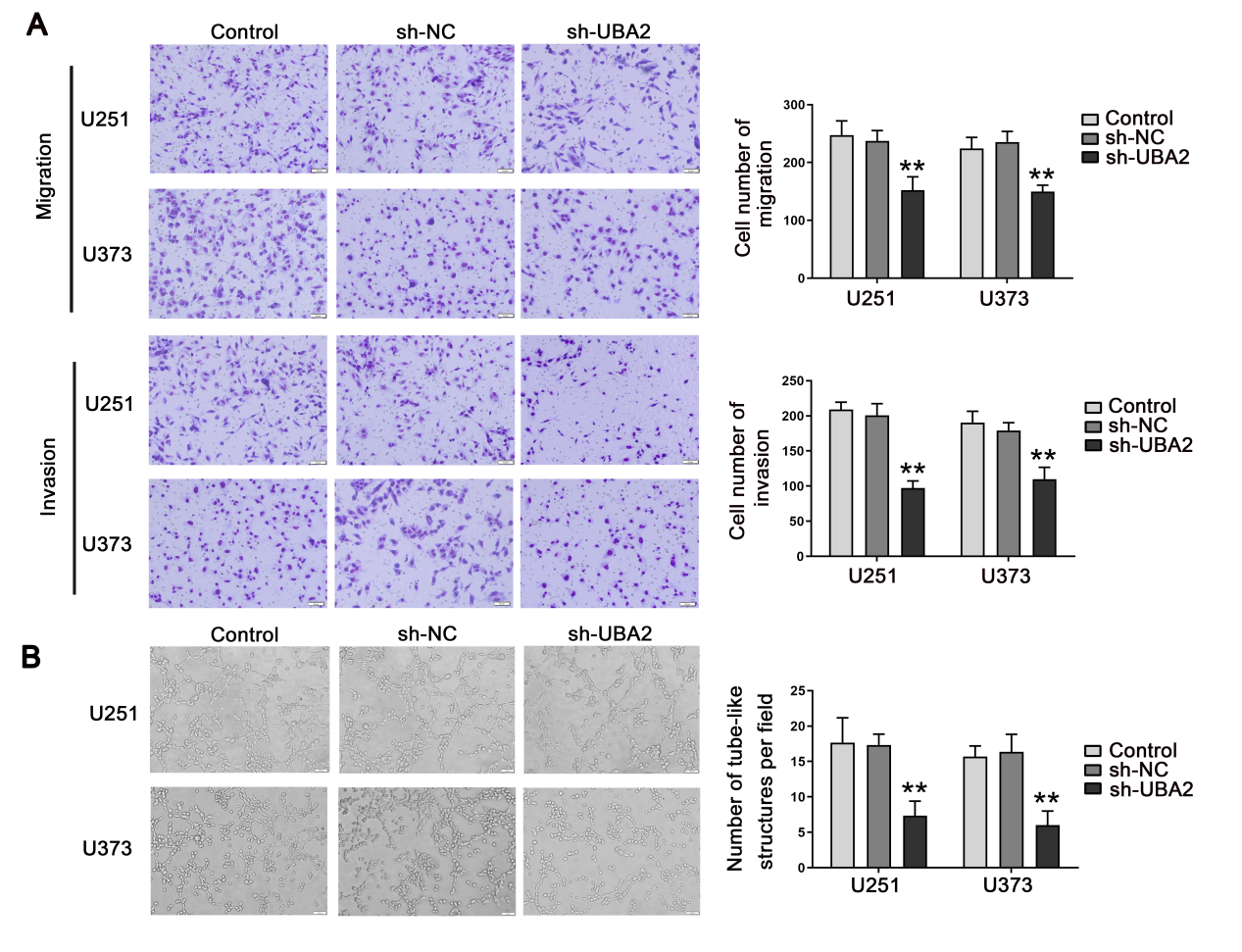
**

**Supplementary Figure 2**. (**A**) Quantification number of migration and invasion cells treated with inhibition of UBA2. (**B**) Three-dimensional cell culture method was used to detect the change of VM in the cells treated with inhibition of RALY on U251 and U373 cells. Representative images and accompanying statistical plots were presented. Data are presented as the mean±SD (n=3 in each group). ***P*<0.01 versus sh-NC group (empty vector); Scale bars represent 50μm. Using one-way analysis of variance for statistical analysis.
