# Supplementary material for: SUMOylation of RALY promotes vasculogenic mimicry in glioma cells via the FOXD1/DKK1 pathway
Source: Cell Biol Toxicol. 2023 Oct 31;39(6):3323–40. doi: 10.1007/s10565-023-09836-3 (PMC10693529; doi:10.1007/s10565-023-09836-3)
Supplement: Supplementary file 3 — Supplementary file3 (DOC 2736 KB) [file 10565_2023_9836_MOESM3_ESM.doc]

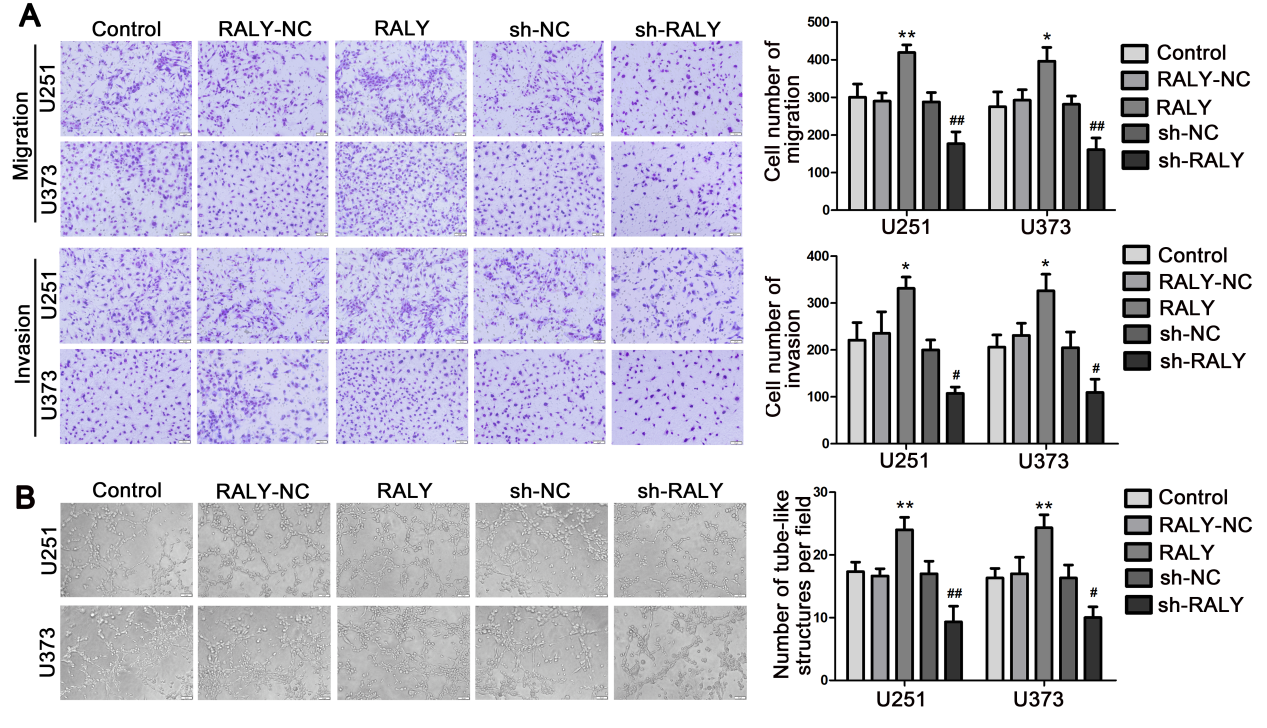


**Supplementary Figure 3.** (**A**) Quantification number of migration and invasion cells treated with altered expression of RALY. (**B**) Three-dimensional cell culture method was used to detect the change of VM in the cells treated with altered expression of RALY on U251 and U373 cells. Representative images and accompanying statistical plots were presented. Data are presented as the mean±SD (n=3 in each group). **P*<0.05, ***P*<0.01 versus RALY-NC group; ^#^*P*<0.05, ^##^*P*<0.01 versus sh-NC group; Scale bars represent 50μm. Using one-way analysis of variance for statistical analysis.
