# Supplementary material for: SUMOylation of RALY promotes vasculogenic mimicry in glioma cells via the FOXD1/DKK1 pathway
Source: Cell Biol Toxicol. 2023 Oct 31;39(6):3323–40. doi: 10.1007/s10565-023-09836-3 (PMC10693529; doi:10.1007/s10565-023-09836-3)
Supplement: Supplementary file 4 — Supplementary file4 (DOC 4194 KB) [file 10565_2023_9836_MOESM4_ESM.doc]

**Supplementary Figure 4.(A)** qRT-PCR was used to detect the expression of FOXD1 mRNA in the cells treated with altered expression of UBA2 and RALY. Each value represents the mean±SD (n=3), **P<0.01 versus sh-NC+RALY-NC group. **(B)** Western blot was used to detect the
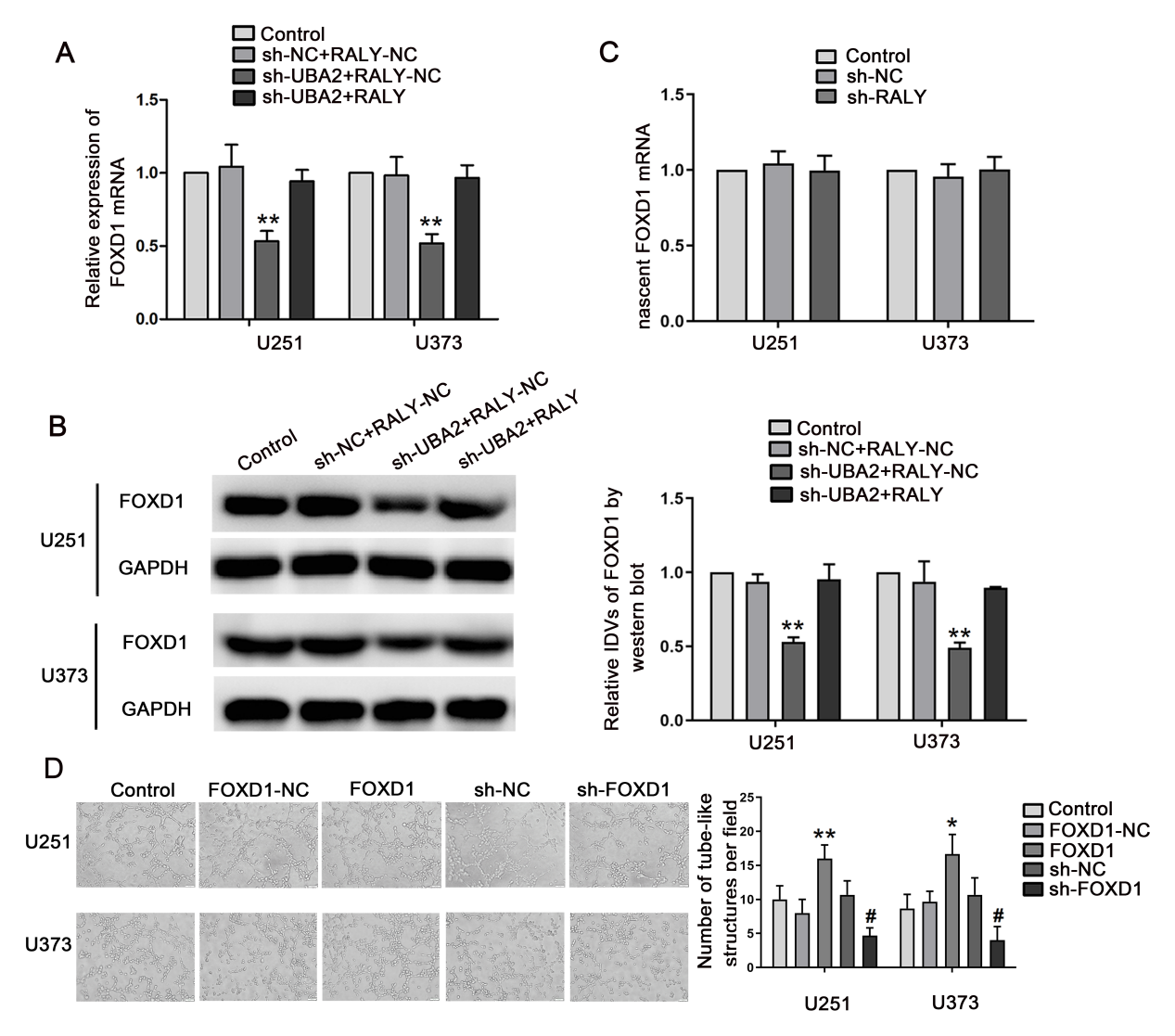
 expression of FOXD1 protein in the cells treated with altered expression of UBA2 and RALY. Representative protein expressions and corresponding IDVs of FOXD1 in U251 and U373 are shown; data are presented as mean±SD (n=3, each group). **P<0.01versus sh-NC+RALY-NC group. **(C)** Click-iT Nascent RNA capture kit (Life Technology) was conducted to label and capture newly synthesized RNA, and nascent FOXD1 mRNA was measured using real-time qPCR. **(D)** Three-dimensional cell culture method was used to detect the change of VM in the cells treated with altered expression of FOXD1 on U251 and U373 cells. Representative images and accompanying statistical plots were presented. Data are presented as the mean±SD (n=3 in each group). **P*<0.05, ***P*<0.01 versus FOXD1-NC group; #*P*<0.05 versus sh-NC group; Scale bars represent 50μm. Using one-way analysis of variance for statistical analysis.
