# Supplementary material for: SUMOylation of RALY promotes vasculogenic mimicry in glioma cells via the FOXD1/DKK1 pathway
Source: Cell Biol Toxicol. 2023 Oct 31;39(6):3323–40. doi: 10.1007/s10565-023-09836-3 (PMC10693529; doi:10.1007/s10565-023-09836-3)
Supplement: Supplementary file 6 — Supplementary file6 (DOC 2511 KB) [file 10565_2023_9836_MOESM6_ESM.doc]

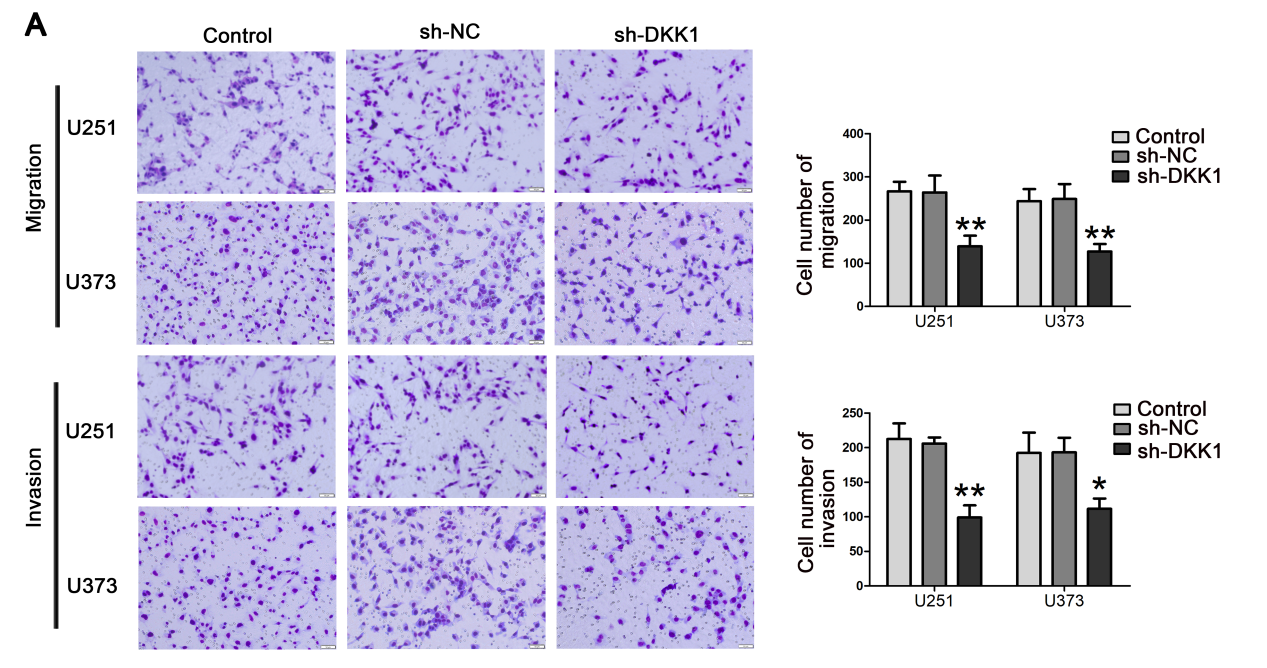


**Supplementary Figure 6**. (**A**) Transwell method was used to detect the change of the capacity for migration and invasion in U251 and U373 cells treated with inhibition of DKK1. Representative images and accompanying statistical plots were presented. Data are presented as the mean±SD (n=3 in each group). **P*<0.05, ***P*<0.01 versus sh-NC group (empty vector); Scale bars represent 50μm. Using one-way analysis of variance for statistical analysis.
