# Supplementary material for: SUMOylation of RALY promotes vasculogenic mimicry in glioma cells via the FOXD1/DKK1 pathway
Source: Cell Biol Toxicol. 2023 Oct 31;39(6):3323–40. doi: 10.1007/s10565-023-09836-3 (PMC10693529; doi:10.1007/s10565-023-09836-3)
Supplement: Supplementary file 7 — Supplementary file7 (DOC 5465 KB) [file 10565_2023_9836_MOESM7_ESM.doc]

**
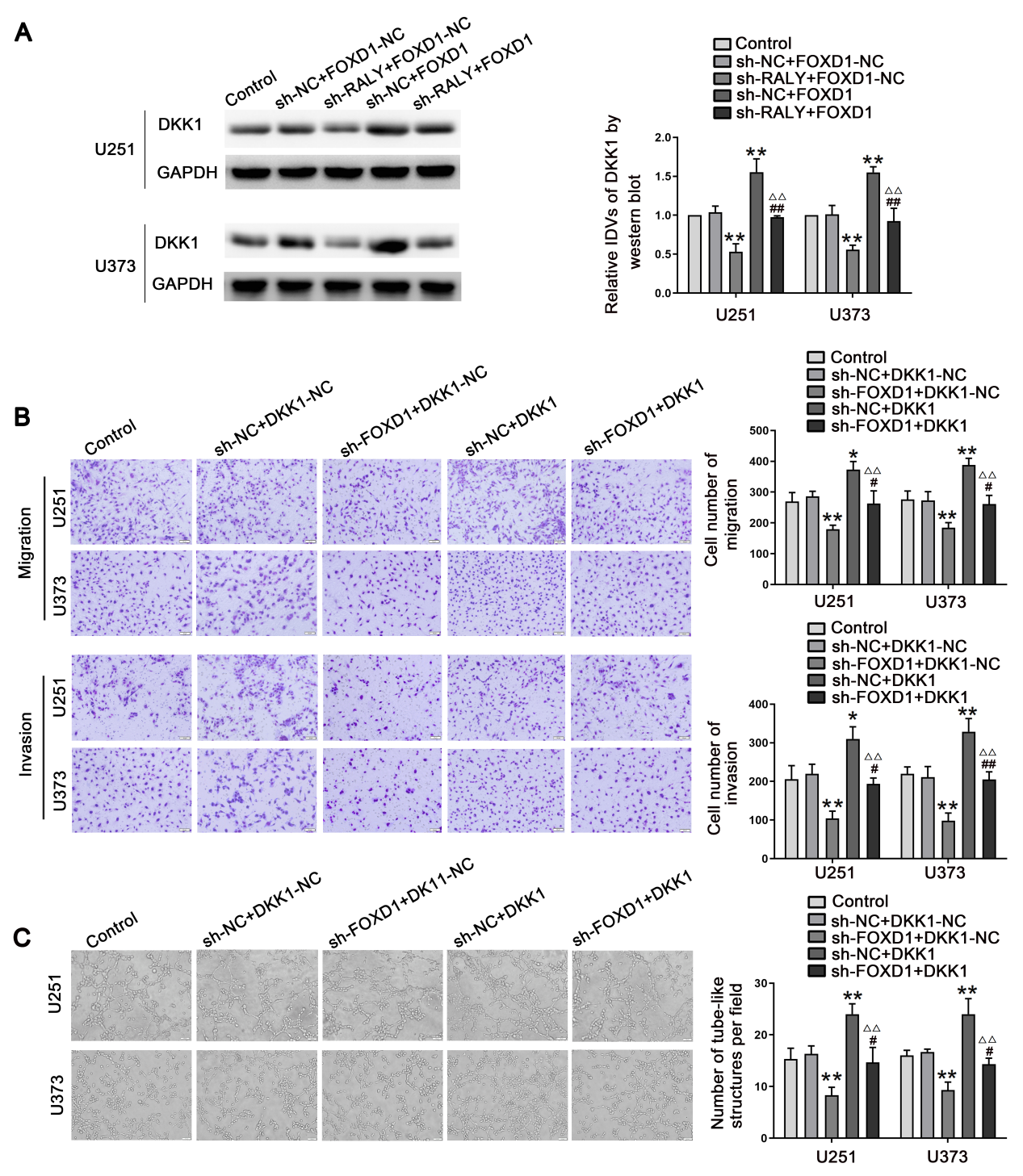
**


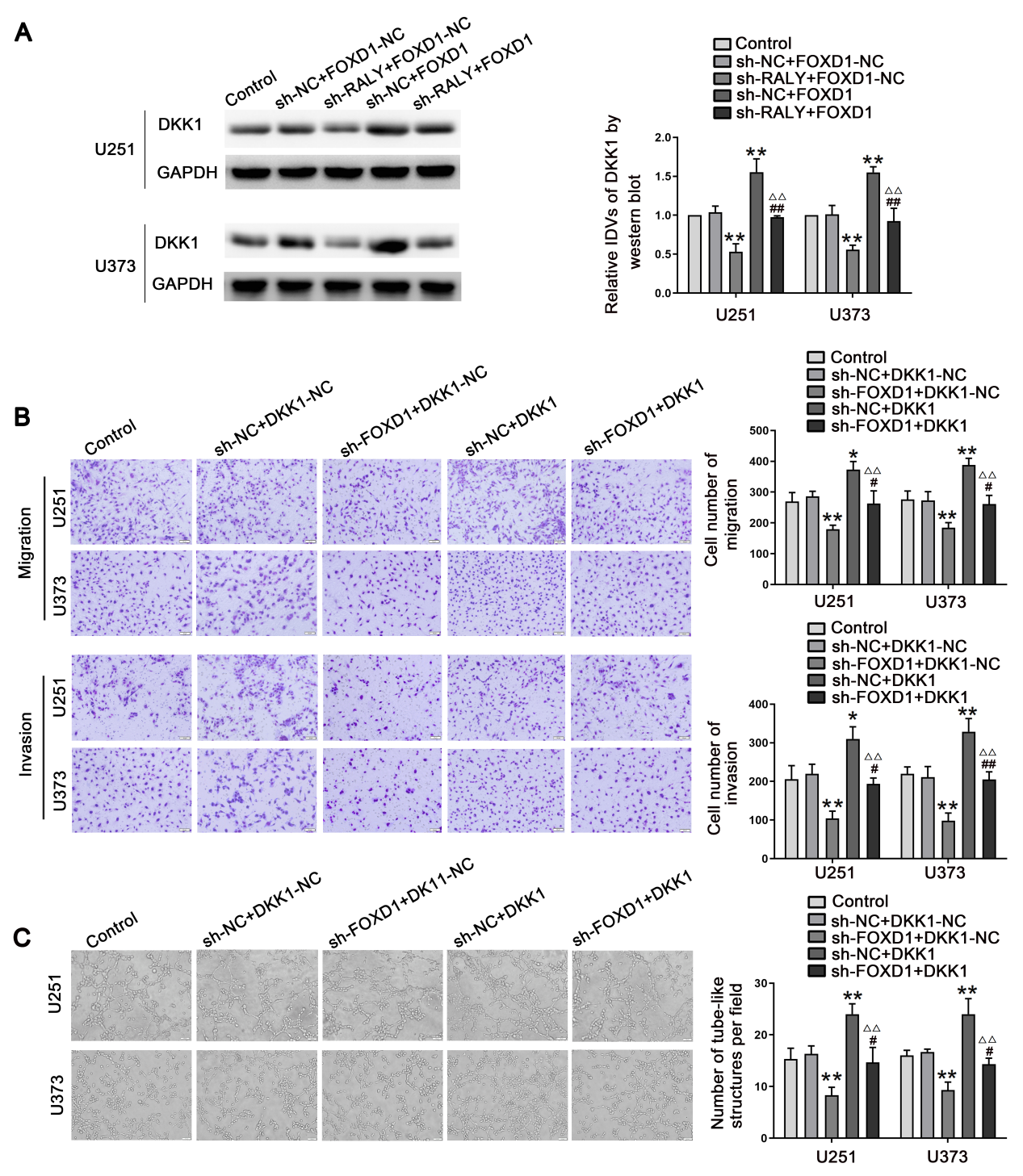
**Supplementary Figure 7.** (**A**) Western blot was used to detect the expression of DKK1 protein in the cells treated with altered expression of RALY and FOXD1. Representative protein expressions and corresponding IDVs of DKK1 in U251 and U373 are shown; data are presented as mean±SD (n=3, each group). (**B**) Quantification number of migration and invasion cells treated with altered expression of FOXD1 and DKK1. (**C**) Three-dimensional cell culture method was used to detect the change of VM in the cells treated with altered expression of FOXD1 and DKK1 on U251 and U373 cells. Representative images and accompanying statistical plots were presented. Data are presented as the mean±SD (n=3 in each group). **P*<0.05, ***P*<0.01 versus sh-NC+DKK1-NC group; *^#^P*<0.05, *^##^P*<0.01 versus sh-FOXD1+DKK1-NC group; ^△△^*P*<0.01 versus sh-NC+DKK1 group; Scale bars represent 50μm. Using one-way analysis of variance for statistical analysis.
