# Supplementary material for: SUMOylation of RALY promotes vasculogenic mimicry in glioma cells via the FOXD1/DKK1 pathway
Source: Cell Biol Toxicol. 2023 Oct 31;39(6):3323–40. doi: 10.1007/s10565-023-09836-3 (PMC10693529; doi:10.1007/s10565-023-09836-3)
Supplement: Supplementary file 8 — Supplementary file8 (DOC 3282 KB) [file 10565_2023_9836_MOESM8_ESM.doc]

**
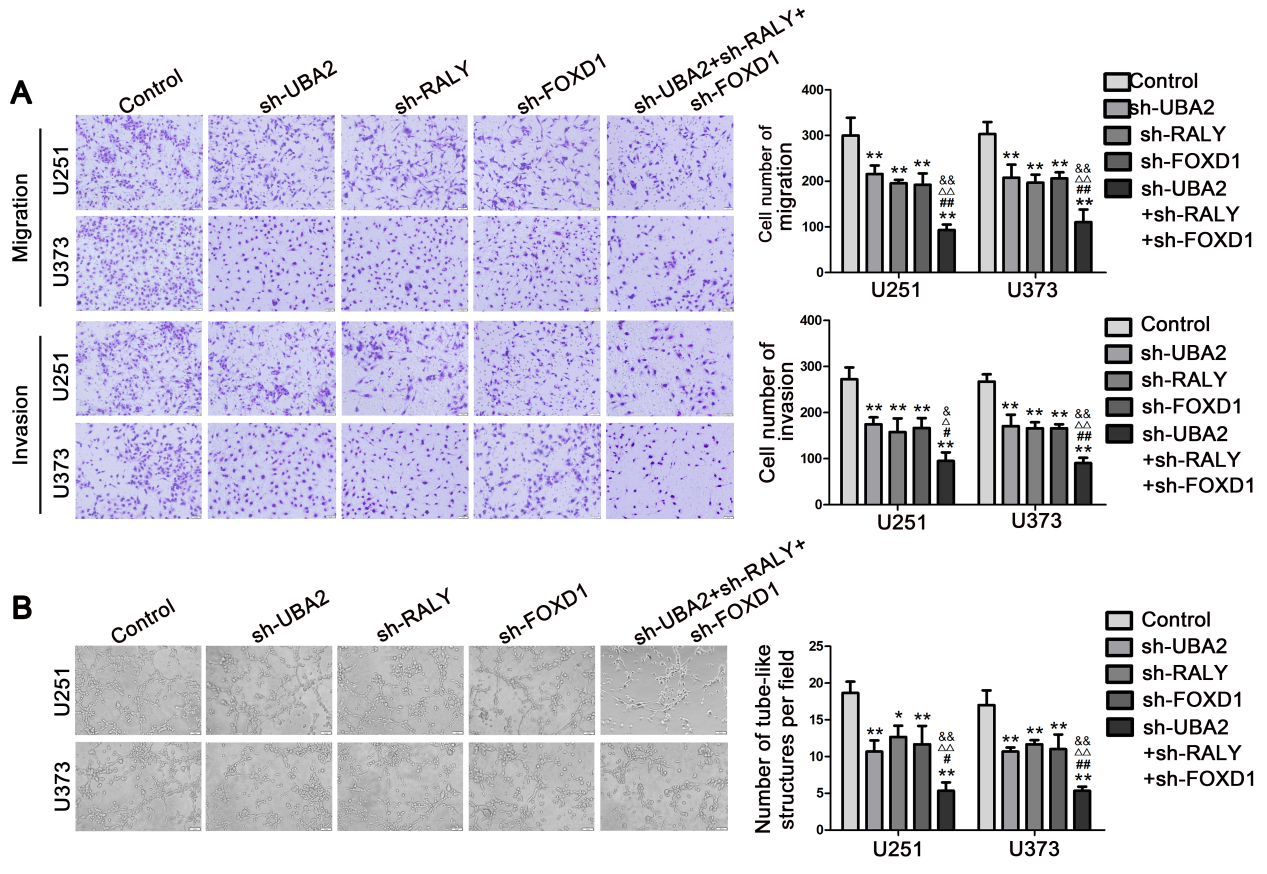
**

**Supplementary Figure 8.** (**A**) Quantification number of migration and invasion cells applied alone and in combination with UBA2 knockdown, RALY knockdown and FOXD1 knockdown. (**B**) Three-dimensional cell culture method was used to detect the change of VM in the cells applied alone and in combination with UBA2 knockdown, RALY knockdown and FOXD1 knockdown on U251 and U373 cells. Representative images and accompanying statistical plots were presented. Data are presented as the mean±SD (n=3 in each group). Scale bars represent 50μm. ******P*<0.05, *******P*<0.01 versus control group; ^#^*P*<0.05, ^##^*P*<0.01 versus sh-UBA2 group; ^△^*P*<0.05, ^△△^*P*<0.01 versus sh-RALY group; ^&^*P*<0.05, ^&&^*P*<0.01 versus sh-FOXD1 group. Using one-way analysis of variance for statistical analysis.
