# Supplementary material for: SUMOylation of RALY promotes vasculogenic mimicry in glioma cells via the FOXD1/DKK1 pathway
Source: Cell Biol Toxicol. 2023 Oct 31;39(6):3323–40. doi: 10.1007/s10565-023-09836-3 (PMC10693529; doi:10.1007/s10565-023-09836-3)
Supplement: Supplementary file 9 — Supplementary file9 (DOCX 11 KB) [file 10565_2023_9836_MOESM9_ESM.docx]

**Table S1**. Primers used for RT-qPCR.

| Primer | Gene | Sequence (5'->3') or Assay ID |
| --- | --- | --- |
| Primer | FOXD1 | F: CACTATGGCCATCCTGCAGC |
|  |  | R:TCGTTGAGCGAGAGGTTGTG |
|  | GAPDH | F: ACAGTCAGCCGCATCTTCTT  R: GCCCAATACGACCAAATCC |
