# Supplementary material for: SUMOylation of RALY promotes vasculogenic mimicry in glioma cells via the FOXD1/DKK1 pathway
Source: Cell Biol Toxicol. 2023 Oct 31;39(6):3323–40. doi: 10.1007/s10565-023-09836-3 (PMC10693529; doi:10.1007/s10565-023-09836-3)
Supplement: Supplementary file 10 — Supplementary file10 (DOCX 11 KB) [file 10565_2023_9836_MOESM10_ESM.docx]

**Table S2**. Primers used for ChIP experiments

| Gene | Binding site or Control | Sequence (5'->3') | Product size (bp) | Annealing temperature (°C) |
| --- | --- | --- | --- | --- |
| DKK1 | PCR1 | F: TCTGCTATAACGCTCGCTGG  R: ACAAAGCCGGGATGGGATTT | 168 | 60.0 |
|  | PCR2 | F: TTTGGGATGGGAAGGACACT  R:CCACCACCAAGTAAAGCCAG | 193 | 58.6 |
