# Supplementary material for: SUMOylation of RALY promotes vasculogenic mimicry in glioma cells via the FOXD1/DKK1 pathway
Source: Cell Biol Toxicol. 2023 Oct 31;39(6):3323–40. doi: 10.1007/s10565-023-09836-3 (PMC10693529; doi:10.1007/s10565-023-09836-3)
Supplement: Supplementary file 11 — Supplementary file11 (DOC 51 KB) [file 10565_2023_9836_MOESM11_ESM.doc]

**Materials and methods**

**Human tissue samples**

Human glioma tissues were collected from patients diagnosed with glioma who undergo neurosurgeries at Shengjing Hospital of China Medical University.NBTs were obtained from fresh autopsy material (donation from individuals who died in a traffic accident and confirmed to be free of any prior pathologically detectable conditions) were used as negative controls. All the tissue samples were immediately frozen in liquid nitrogen after surgical resection for the ensuing experiments. The study was approved by the Ethics Committee of Shengjing Hospital of China Medical University, and all patients voluntarily signed informed Consent. Glioma specimens were divided into two groups: grade I–II glioma group (low-grade glioma tissues) and grade III–IV glioma group (high-grade glioma tissues) according to the 2007 WHO classification of tumors in the central nervous system by neuropathologists.

**Quantitative real-time PCR (qRT-PCR)**

Total RNA was extracted from the glioma specimens, normal brain tissues and cells (HA, U251 and U373) using Trizol reagent (Life Technologies Corporation, Carlsbad, CA). RNA concentration and quality were determined via 260/280 nm absorbance with Nanodrop Spectrophotometer (ND-100, Thermo, Waltham, MA). One-Step SYBR Prime-Script RT-PCR Kit (TakaraBio, Inc., Japan) was used to detect the expression of FOXD1 and GAPDH in 7500 Fast RT-PCR System (Applied Biosystems, USA). Expressions were normalized using GAPDH as the endogenous controls, and the relative quantification (2^-△△Ct^) method was used for fold-change calculation. The primers for each PCR set are listed in Table S1.

**Cell culture and transfections**

Human U251 and U373 glioma cell lines were purchased from Shanghai Institutes for Biological Sciences Cell Resource Center. The human astrocytes (HA) cells were purchased from Shanghai Zeye Biotechnology. All cells were cultured in a humidified incubatorat 37 °C with 5% CO2. Cell transfection was performed using Lipofectamine 3000 reagent (Life Technologies Corporation, Carlsbad, CA) according to the manufacturer’s instructions. Short hairpin RNA against UBE2I (sh-UBE2I), short hairpin RNA against RALY (sh-RALY), short hairpin RNA against FOXD1 (sh-FOXD1), RALY full length plasmid (HA-RALY-WT), FOXD1 full length plasmid (FOXD1) and their respectivenon-targeting sequence (negative control, NC) were synthesized (GenePharma, Shanghai, China). Short hairpin RNA against SENP1 (sh-SENP1), short hairpin RNA against DKK1 (sh-DKK1), SENP1 full length plasmid (Myc-SENP1), RALY full length plasmid (RALY-Mut (K175R)) and their respective non-targeting sequence (negative control, NC) were synthesized (GeneChem, Shanghai, China). UBE2I full-length plasmid (Flag-UBE2I), SUMO1 full-length plasmid (His-SUMO1) and their respective non-targeting sequence (negative control, NC) were synthesized (JTS, Beijing, China). Plasmids carrying non-targeting sequence were used as negative control, and the transfection efficacy was assessed by qRT-PCR or Western blotting.

**Western blot**

The proteins either from whole cell lysates in the SDS-lysis buffer or derived from immunoprecipitations, were run on SDS-PAGE gels and electrophoretically transferred to PVDF membranes, which were blocked by Tween-Tris-buffered saline (TTBS) for 2h at room temperature and then incubated with primary antibodies as follows: RALY (abcam, UK), FOXD1 (Santa Cruz, USA), DKK1 (Santa Cruz, USA), HA ( Santa Cruz, USA), His (Santa Cruz, USA), Myc (Proteintech, USA), Flag (Proteintech, USA), GAPDH (Proteintech, USA). After incubating with secondary antibodies for 2h at room temperature, immunoblots were developed with enhanced chemiluminescence (ECL kit, Beyotime, Shanghai, China) and blot bands were scanned using ChemImager 5500 V2.03 software (Alpha Innotech, San Leandro, CA) according to the manufacturer’s protocol. The relative integrated density values (IDVs) were calculated using Fluor Chen 2.0 software based on GAPDH as an internal control.

**Cell migration and invasion assay**

Cells (2 × 10^5^) were suspended in 100 μl serum-free medium at a density of 10^5^/ml and seeded into the upper chamber (or precoated with 80 μl of Matrigel solution (BD, Franklin Lakes, NJ, USA) for cell invasion assay).The bottom-well were filled with 600 μl of 10% FBS medium. Cells that had migrated or invaded to the lower surface of the membrane were fixed with methanol and glacial acetic acid, and then stained with 20% Giemsaand. Five random fields were chosen to count cells and take photographs.

**Cells VM formation assays**

Each well in the 96-well culture plate was covered with 100μl of Matrigel solution and incubated for 30 min in the incubator at 37℃. The cells were resuspended and seeded with serum-free medium at a density of 3 × 10^5^ cells per wellonto the surface of matrigel and incubated at 37 °C for 8 h. The cell vascular structures were observed and pictures were taken with an inverted microscope (Olympus, Tokyo, Japan).

**Immunofluorescent staining**

Cells were fixed with 4% paraformaldehyde for 30 min and then permeabilized with 0.2% Triton X-100 for 1 h, and then incubated in the primary antibody at 4 °C overnight. After washing three times with PBS and then incubated with fluorescent dye-conjugated secondary antibody diluted in 5% goat serum in PBS for 2h at room temperature. The nuclei were then counterstained with DAPI for 5 min. The staining was visualized using confocal microscopy.

**RNA immunoprecipitation assay (RIP)**

The RNAs bound to RALY were detected by RNA binding protein immunoprecipitation (RIP) assay. RIP assays were performed according to the manufacture’s protocol by using EZ-Magna RNA-binding protein immunoprecipitation kit (Millipore, USA). The whole-cell lysate was incubated with RIP buffer containing magnetic beads conjugated with anti-IgG (as negative control) or human anti-RALY antibody. Samples were incubated with Proteinase K and immunoprecipitated RNA was isolated. Furthermore, RNA was purified from RNA–protein complexes and analyzed by qRT-PCR.

**RNA stability measurement**

After transfected with sh-NC, sh-RALY, RALY-WT or RALY-K175R, 2 μg/ml Actinomycin D was added to inhibit de novo RNA synthesis. Total RNA was extracted at different times and FOXD1 mRNA expression was measured by qRT-PCR. The half-life of FOXD1 mRNA was determined by its level reach 50% of the RNA level before the addition of actinomycin D.

**Protein half life assay**

HA-RALY-WT was stably expressed in U251 cells transduced with sh-NC or sh-UBA2. cycloheximide (50μg/ml) was added into the transfected cells for the time indicated in the results. The cell extracts at each time point were separated by SDS PAGE and then measured by western blot. The half-life of RALY was determined by its level reach 50% of the protein levels at 0h.

**Chromatin immunoprecipitation (ChIP) assay**

According to the manufacturer’s protocol, ChIP assay was conducted using Simple ChIP Enzymatic Chromatin IP Kit (Cell Signaling Technology, Danvers, Massachusetts, USA). In brief, glioma cells were crosslinked with 1% formaldehyde and collected in lysis buffer. Immunoprecipitation samples were incubated with normal rabbit IgG or anti-FOXD1 antibody. DNA crosslinks were reversed by 5 mol/l NaCl and proteinase K at 65 °C for 2 h, and then purified. Immunoprecipitated DNA was amplified by PCR using their specific primers. The primers for each PCR set, the sizes of PCR products, and annealing temperatures are listed in Table S2.

**CD34-periodic acid-schiff (PAS) dual-staining**

CD34-PAS is used for qualitative and quantitative analyses of VM in tissue sections of nude mice xenografts. 5μm formalin-fixed and paraffin-embedded tissue specimens were dewaxed in xylene, hydrated in gradient ethanol, and boiled in EDTA antigen-unmasking solution. After cooling to room temperature, the specimens were incubated with peroxide, and stained with CD34 primary monoclonal antibody (1:50, Proteintech, Rosemont, IL) overnight at 4℃. After incubation with secondary antibody at room temperature for 10 min, the specimens were treated with DAB kit (Fuzhou MaiXin Biotech, China). VM density was counted under microscope in five random fields.

**Tumor xenografts in nude mice**

For the in vivo study, the stably transfected cells (U251 and U373) were divided into five groups: Control, sh-UBA2, sh-RALY, sh-FOXD1, sh-UBA2+sh-RALY+sh-FOXD1. Each nude mouse was subcutaneously injected with a suspension of 3×10^5^ cells in a 100μl volume into the right flanks of mice. The tumor volume was measured every 4 days when the tumors were clearly identified and the volume was calculated according to the formula: volume (mm^3^)=length×width^2^/2. All mice were sacrificed on the 40th day and the tumors were isolated, photographed and weighed. For survival analysis in orthotopic inoculations , 3×10^5^cells were implanted into the right striatum of mice stereotactically. The number of survived nude mice was recored, and survival analysis was performed using Kaplan–Meier survival curve. All animal studies were conducted with the Care and Use of Laboratory Animals and protocols approved by the Animal Care Committee of Shengjing Hospital.

**Statistical analysis**

All data are presented as mean±SD. All statistical analyses were evaluated by SPSS 18.0 statistical software (IBM, New York, NY) with the Student’s *t*-test (two tailed) or one-way analysis of variance. Survival analysis was evaluated using the Kaplan-Meier method and assessed using the log-rank test. *P*<0.05 was considered statistically significant.
